# Integrated analysis of global proteome, phosphoproteome, and glycoproteome enables complementary interpretation of disease-related protein networks

**DOI:** 10.1038/srep18189

**Published:** 2015-12-11

**Authors:** Jong-Moon Park, Ji-Hwan Park, Dong-Gi Mun, Jingi Bae, Jae Hun Jung, Seunghoon Back, Hangyeore Lee, Hokeun Kim, Hee-Jung Jung, Hark Kyun Kim, Hookeun Lee, Kwang Pyo Kim, Daehee Hwang, Sang-Won Lee

**Affiliations:** 1 Department of Pharmaceutics, College of Pharmacy, Gachon University, Incheon 406-799, Republic of Korea; 2 Department of Chemical Engineering, POSTECH, Pohang 790-784, Republic of Korea; 3 Department of Chemistry, Research Institute for Natural Sciences, Korea University, Seoul 136-701, Republic of Korea; 4 Department of Applied Chemistry, College of Applied Sciences, Kyung Hee University, Yong-in 446-701, Republic of Korea; 5 National Cancer Center, Goyang 410-769, Republic of Korea; 6 Department of New Biology and Center for Plant Aging Research, Institute for Basic Science, DGIST, Daegu 711-873, Republic of Korea

## Abstract

Multi-dimensional proteomic analyses provide different layers of protein information, including protein abundance and post-translational modifications. Here, we report an integrated analysis of protein expression, phosphorylation, and N-glycosylation by serial enrichments of phosphorylation and N-glycosylation (SEPG) from the same tissue samples. On average, the SEPG identified 142,106 unmodified peptides of 8,625 protein groups, 18,846 phosphopeptides (15,647 phosphosites), and 4,019 N-glycopeptides (2,634 N-glycosites) in tumor and adjacent normal tissues from three gastric cancer patients. The combined analysis of these data showed that the integrated analysis additively improved the coverages of gastric cancer-related protein networks; phosphoproteome and N-glycoproteome captured predominantly low abundant signal proteins, and membranous or secreted proteins, respectively, while global proteome provided abundances for general population of the proteome. Therefore, our results demonstrate that the SEPG can serve as an effective approach for multi-dimensional proteome analyses, and the holistic profiles of protein expression and PTMs enabled improved interpretation of disease-related networks by providing complementary information.

Multiple types of information, including protein abundances and various post-translational modifications (PTMs), such as phosphorylation or glycosylation, are required to understand activities of multilayered cellular protein networks. Comparison of protein abundances between two conditions reveals alterations in activities of cellular protein networks that can be reflected by protein abundances. Also, phosphorylation levels represent activities of signaling pathways in cellular protein networks. The levels of other PTMs, such as ubiquitination or acetylation, represent the states of the proteins, thereby reflecting activities of cellular protein networks associated with such states of the proteins. Thus, multi-dimensional proteomic analyses have been employed to acquire multiple types of protein data to decode diverse activities of cellular protein networks.

Mass spectrometry (MS)-based global proteome analysis has provided abundance data only for a limited number of proteins, compared to transcriptomic analysis, to understand activities of cellular protein networks. Moreover, due to relatively low abundances of PTMs, the enrichment of peptides with PTMs is commonly employed prior to liquid chromatography-tandem mass spectrometry (LC-MS/MS) analysis, which requires larger amounts of samples for profiling the peptides with PTMs, compared to transcriptomic analysis. However, recent advances in extensive fractionation, high-resolution peptide separation, and high performance mass spectrometry significantly improved the proteome size as to be comparable to the transcriptome size detected by mRNA-sequencing. For example, Branca *et al.* measured the abundances of 13,078 proteins from human A431 cells using high-resolution isoelectric focusing prefractionation^1^. Moreover, serial enrichment methods of peptides with PTMs from the same sample have been developed to significantly reduce the sample amount required. For example, Mertins *et al.* developed serial enrichments of different post-translational modifications (SEPTM) for an integrated analysis of protein abundance, phosphorylation, ubiquitination, and acetylation from the same sample^2^. The SEPTM method enabled quantitative analysis of about 8,000 proteins, and 20,000 phosphorylation, 15,000 ubiquitination and 3,000 acetylation sites per experiment in human leukemia cells, permitting a holistic view of cellular signal pathways.

Such advances in MS-based proteomic analysis have facilitated multi-dimensional proteomic analyses for understanding activities of multilayered protein networks. Swaney *et al.* performed an integrated analysis of phosphoproteome and ubiquitinated proteomes in *Saccharomyces cerevisiae* and identified 466 proteins with 2,100 phosphorylation sites co-occurring with 2,189 ubiquitinated sites^3^. Using these data, they found interplays between the signaling networks represented by phosphorylation and ubiquitination and identified phosphorylation sites regulating ubiquitin-dependent protein degradation based on the interplays. Likewise, many of these studies focused on cellular protein networks commonly represented by different types of proteomic data. For example, the integrated analysis of phosphoproteome and lysine-acetylome in *Mycoplasma pneumonia* revealed interplays of the protein networks represented by the two PTMs^4^. These studies have showed that multiple types of proteome profiles can add both depth of detection and complementary information. However, how different types of proteomic data complementarily improved interpretation of cellular protein networks has not been systematically examined.

Here, we present a MS-based method for multi-dimensional proteomic analyses of protein abundance, phosphorylation, and N-glycosylation by serial enrichments of phosphorylation and N-glycosylation from the same tissue samples. In this method, isobaric tag for relative and absolute quantitation (iTRAQ) labeled peptides from human gastric cancer and adjacent normal tissues of a patient were subjected to fractionation by mid-pH reverse phase LC (RPLC) and then to the immobilized-metal affinity chromatography (IMAC) method for the phosphopeptide enrichment, followed by a filter aided capture and elution (FACE) for the N-glycopeptide enrichment. Our serial enrichment method is a variation of the SEPTM method developed by Mertins *et al.*^2^ to add the enrichment of N-glycosylated peptides without the enrichment of ubiquitinated and acetylated peptides. LC-MS/MS analysis provided global proteome, phosphoproteome, and N-glycoproteome for the cancer and normal tissues from three patients. The combined analysis of these data demonstrated that different types of data provided complementary coverages of gastric cancer-related protein networks, thereby facilitating improved interpretation of the networks.

## Methods

### Tissue collection

Gastric tissues samples (cancer and adjacent normal tissues) were collected from three patients with gastric cancer who underwent gastrectomies at Asan Medical Center and Chonnam University Hwasun Hospital, which are members of National Biobank of Korea (2013-7 and 07SA2013010-001) (Supplementary Table S1). The methods for tissue processing were approved in accordance with the experimental protocols by the National Biobank of Korea. Institutional review board (AMC-2012-0576, NCCNCS-120581)-approved informed consents were signed by all subjects. There were 2 males (66.7%) and median age was 44 years (40–45). All three tumors were diffuse type in histology and microsatellite-stable. At the time of diagnosis, two patients were stage IIB and one patient was at stage IV. No patients received prior chemotherapy or radiotherapy. After gross examination, non-necrotic portions were excised from resected tumor specimens by pathologists and immersed in liquid nitrogen within 30 minutes after resection. Tissue lysis was performed for tumor-rich area of each cancer tissue that was identified by the light microscopic evaluation of hematoxylin and eosin-stained top slides. Adjacent normal tissues were taken from the luminal side of the gastrectomy specimen, at least 2cm apart from the tumor border, and immersed in liquid nitrogen at the same time with the tumor tissues of the same patients.

### Serial enrichments of phosphorylation and N-glycosylation (SEPG) from the same tissue sample

**Figure 1:**
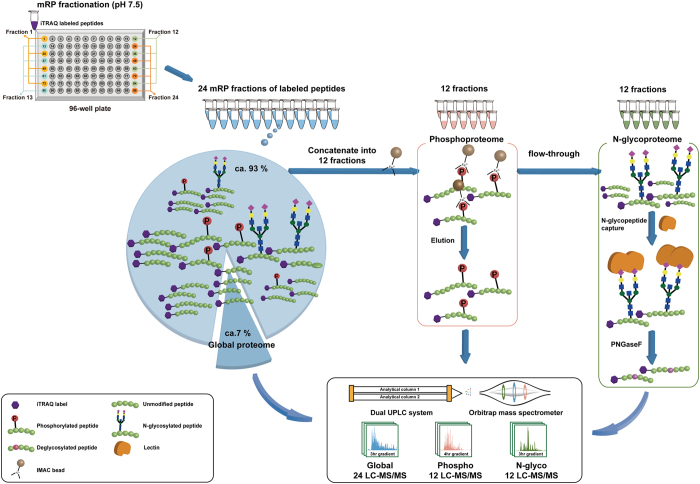
Overall workflow in the SEPG method for integrated profiling of global proteome, phosphoproteome, and N-glycoproteome. First, iTRAQ-labeled peptides were fractionated into 24 fractions using mRP fractionation on the 96-well plate as schematically described (top left). For each of 24 fractions, global profiling was then performed, and the remaining 24 fractions were further concatenated into 12 fractions. For each of the 12 fractions, IMAC enrichment was performed, followed by phosphoproteome profiling. Finally, for the flow-throughs of the 12 fractions from the IMAC experiments, N-glycopeptide enrichment was performed, followed by N-glycoproteome profiling.

We developed an integrated method for profiling global proteome, phosphoproteome, and N-glycoproteome by SEPG from the same tissue samples (Fig. 1). In this method, we used a FASP for protein digestion. The method permits to use sodium dodecyl sulfate, a potent detergent for solubilizing hydrophobic membranous proteins, and thereby to produce ‘universal’ proteome from nucleus to membrane and extracellular matrix^5^, which can facilitate the integrated multi-dimensional proteomic analyses. The peptides obtained from the pair of tumor and adjacent normal tissues of a gastric cancer patient were then individually double-labeled with 4-plex iTRAQ reagents (Supplementary Methods).

We next used a mid-pH RPLC fractionation (mRP fractionation) from which the initial 96 fractions of the iTRAQ-labeled peptides were noncontiguously pooled into 24 fractions (Fig. 1; Supplementary Methods). In the mRP fractionation, we used triethylammonium bicarbonate (TEAB) solvents (pH 7.5), instead of the conventional ammonium formate solvents (pH 10)^6^. TEAB was chosen for three reasons: better compatibility with iTRAQ labeling, high volatility, and less detrimental effects on the separation column. As iTRAQ labeling uses TEAB as a buffer, we concentrated the iTRAQ-labeled peptide solution down to a small volume (i.e. 200 μL), rather than completely drying, and directly injected for the subsequent mRP fractionation. Also, due to the high volatility of TEAB, mRP fractions were dried and directly used in the subsequent LC-MS/MS or IMAC experiments. Finally, the use of TEAB for the mRP fractionation showed no degradation to the C18 column over a period of 1 year usage and up to more than 100 fractionation experiments, whereas the ammonium formate in the buffers was reported to have detrimental effects on column^7^.

Next, individual mRP fractions (10 μg, ca. 7%) were subjected to LC-MS/MS experiments to profile global proteome. The rest peptides were concatenated into 12 fractions (Supplementary Methods) each of which was then subjected to the phosphopeptide enrichment experiments, and the flow-throughs in the IMAC experiments were used for the N-glycopeptide enrichment experiments^8^,^9^ (Fig. 1). The entire procedure of the SEPG developed for the integrated multi-dimensional proteome analyses was performed without a single offline desalting step.

The combined use of two orthogonal dimensions (i.e. mid-pH RP and low-pH RP)^10^ and the noncontiguous pooling strategy effectively spread the complex peptide mixture into peptide fractions of even complexity as evidenced by uniform distributions of identified peptides over the RPLC retention time across the 24 fraction LC-MS/MS experiments (Supplementary Fig. S1). The effective fractionation was also supported by the observations of ca. 80% and 88% of the global and phosphopeptides, respectively, being uniquely identified from the individual fractions on average (Supplementary Fig. S2). Relatively small portion (ca. 54%) of N-glycopeptides being uniquely identified from the individual fraction (Supplementary Fig. S2) may reflect the inherent complex nature of protein glycosylation, as a single glycosylation site was previously observed to be occupied by many structurally different glycans^11^. These N-glycosylated peptides of different glycans will be fractionated into different fractions, but will become the same deglycosylated peptides (with Asn → Asp) after deglycosylation by PNGase F during FACE enrichment experiments, resulting in the same deglycosylated peptides in different fractions.

### Identification of differentially expressed proteins, phosphoproteins and glycoproteins

The raw data used in this study were deposited into PRIDE database (dataset identifier PXD003115)^12^,^13^. iTRAQ intensities of the 143,020, 140,165, and 143,133 aligned peptides (on average, 142,106 peptides; Supplementary Table S2a–c) were normalized using the quantile normalization method^14^. Log_2_-fold-changes of the proteins between gastric cancer and adjacent normal tissues were estimated using the linear-programming method, as previously described^15^, based on the bipartite graph representing protein groups. Of these quantified proteins, the proteins more than two non-redundant peptides were chosen for the following analyses. First, a statistical hypothesis test for the log_2_-fold-changes was used to identify differentially expressed proteins (DEPs)^16^. An empirical null distribution was estimated by applying Gaussian kernel density estimation method to log_2_-fold-changes obtained after performing all possible permutations of 114–117 labels^17^. The proteins with *P* < 0.05 at least in one of the three tissue pairs were identified as the DEPs. Second, for 19,421, 16,973, and 20,144 phosphopeptides and 4,405, 4,161, and 3,491 N-glycopeptides (on average, 18,846 phosphopeptides and 4,019 N-glycopeptides; Supplementary Table S2a–c), the quantile normalization and the same statistical method was used at the peptide level. The empirical distribution and *P*-values were estimated for the log_2_-fold-changes of the peptides. Differentially phosphorylated proteins (DPPs) and differentially N-glycosylated proteins (DGPs) were identified as the proteins that contained the unique phosphopeptides and N-glycopeptides with *P* < 0.05, or the unique peptides detected exclusively in either the gastric tissue or the normal tissue. Finally, the enrichment analyses of gene ontology biological processes (GOBPs), gene ontology cellular components (GOCCs), and Kyoto encyclopedia of genes and genomes (KEGG) pathways^18^ were performed for the DEPs, DPPs, and DGPs using ConsensusPathDB^19^ software. The GOBPs, GOCCs, and KEGG pathways represented by the DEPs, DPPs, and DGPs were identified as the ones with *P* < 0.1 (a default cutoff). Alternatively, *Z* score was also computed as *Z* = N^−1^ (1 − *P*), where N^−1^(∙) is the inverse standard normal distribution and *P* is the enrichment *P*-value.

### Five types of cellular protein networks

We categorized the cellular protein networks in the KEGG pathway database to the following five groups as shown in Supplementary Table S3: 1) metabolic networks, 2) cellular signaling networks, 3) cell cycle, DNA, and RNA processing networks, 4) protein homeostasis networks, and 5) cellular and organic interaction networks. The proteins in the cellular signaling networks were further grouped into receptor ligands, receptors, kinases/phosphatases, and transcription factors based on the gene ontology molecular functions (‘Receptor binding’, ‘Receptor activity’, ‘Kinase activity’, ‘Phosphatase activity’, and ‘Nucleic acid binding transcription factor activity’, respectively).

### Construction of cellular network models for complementary coverages

The 191,822 protein-protein interactions (PPIs) for the 16,382 proteins were obtained from the Biological General Repository for Interaction Datasets (BioGRID)^20^, CCSB interactome database (CCSB)^21^,^22^,^23^,^24^, the Human Protein Reference Database (HPRD)^25^, the IntAct molecular interaction database (IntAct)^26^, the Molecular INTeraction database (MINT)^27^, and the Database of Interacting Proteins (DIP)^28^, as shown in Supplementary Table S4. The networks for the CC1-3 were constructed by adding the PPIs to the DEPs, DPPs, and DGPs to the interactions in the corresponding KEGG pathways.

### Identification of hub-like molecules and nodes with large clustering coefficient

Using the PPIs described above, we computed the number of interactors (degree) and clustering coefficient for each protein in the following sets: 1) DEPs, 2) DEP + DPPs, 3) DEP + DGPs, and 4) DEP + DPP + DGPs. For each set, the empirical distribution of the degree was estimated by randomly sampling the same number of proteins from the whole proteome with PPIs 1,000 times and then by computing the degrees for the randomly sampled proteins. Based on the empirical distribution, the *P*-value for each protein was computed using the right-sided test, and the proteins with *P* < 0.05 were identified as the hub-like molecules. The same procedure was used to compute *P*-values for clustering coefficients and to select the nodes with large clustering coefficients (*P* < 0.05).

### TF and kinase enrichment analysis

To identify key TFs regulating the DEPs, 304,217 protein-DNA interactions (PDIs) were first collected from the human transcriptional regulation interactions database (HTRIdb)^29^, transcriptional regulatory element database (TRED)^30^, Amadeus^31^, the molecular signatures DB (MSigDB)^32^, EdgeExpress database (EEDB)^33^, a database of regulatory information for human bZIP transcription factors (bZIPDB)^34^, and MetaCore™ (GeneGo, St. Joseph, MI, USA) shown in Supplementary Table S5. For each TF, the number of targets in the DEPs was calculated using the PDI data, and *P*-value for the number of targets was then computed based on the hypergeometric distribution by applying Fisher’s exact test. Of the TFs with *P* < 0.05, the TFs belonging to the DEPs or DPPs were chosen as the key regulators. To identify key kinases regulating the DPPs, 5,563 kinase-substrate interaction data were also collected from PhosphoSitePlus®^35^, Phospho.ELM^36^, and PhosphoPOINT^37^ (Supplementary Table S6). The same method was applied to the kinase-substrate interaction data and the DPPs. Of the kinases with *P* < 0.05, the kinases belonging to the DEPs or DPPs were selected as key kinases. For the following two groups, we calculated the network density (*D*) as *E*/_*n*_C_2_, where *E* and *n* is the number of interactions and nodes in the network, respectively, and _*n*_C_2_ is the number of possible interactions in the network: 1) 17 key TFs and kinases belonging to the DEPs, and 2) 30 key TFs and kinases belonging to the DEPs or DPPs. For the network density of each group, we estimated an empirical distribution of *D* by randomly sampling the same number of proteins with the group from the 1,320 TFs and kinases with PPIs 100,000 times and then computed *P*-values of the observed *D* based on the empirical distribution using the right-sided test.

## Results

### Multi-dimensional proteomes obtained by the SEPG method

**Figure 2:**
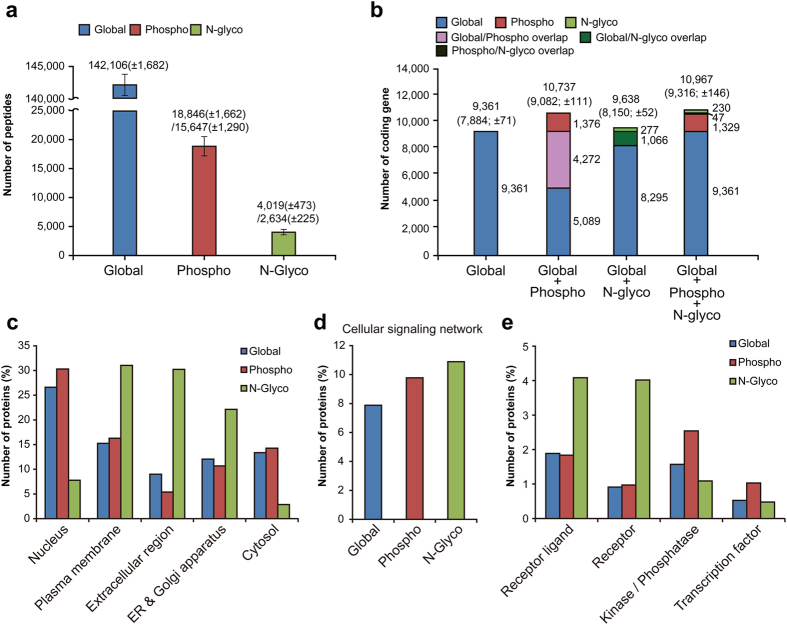
Multi-dimensional proteomes measured by the SEPG method. (**a**) Averaged numbers of identified peptides from global proteomes, phosphoproteomes, and N-glycoproteomes for the three gastric cancer patients. Data were shown as means ± SD. (**b**) Numbers of protein coding genes measured by the three proteomes. Colored stacked bar graphs showed incremental identifications of the genes by the indicated multi-dimensional proteomes (see color legend). (**c–e**) Percentages of proteins measured by the three proteomes that were localized in the indicated five major organelles based on their gene ontology cellular components (**c**) and that were involved in the cellular signaling networks in the KEGG pathway database (**d**,**e**) Percentages of proteins involved in the cellular signaling networks that belonged to the indicated four groups of signaling molecules.

To understand the characteristics of the multi-dimensional proteomes measured by the SEPG method, we first identified the peptides from the three proteomes using the Uniprot protein sequences (May, 2013; 90,191 entries) in the target-decoy setting by the MS-GF+ (v9387) search engine^38^ (**Methods**). For 24 global LC-MS/MS datasets, on average, we identified 142,106 non-redundant peptides (8,625 protein groups) for the three patients (Supplementary Table S2a-c, S7a-c and Fig. 2a). We also identified 18,846 phosphopeptides (15,647 phosphosites) and 4,019 N-glycopeptides (2,634 N-glycosites), on average (Supplementary Table S2a-c, S7a-c and Fig. 2a), belonging to 4,560 and 1,140 protein groups, respectively. Importantly, the three proteomes increased cumulatively the coverage of the cellular proteome (Fig. 2b). Global proteomes detected from the three patients were mapped to 9,361protein coding genes (on average, 7,884 protein coding genes per patient), and phosphoproteomes were mapped to 5,648 genes, 1,376 of which were additionally identified by phosphoproteome analysis. Furthermore, N-glycoproteomes were mapped to 1,343 genes, 277 of which were identified uniquely by N-glycoproteome analysis.

This incremental protein identification by the three proteomes suggests complementary coverages of cellular proteome networks. Some PTMs can occur predominantly in particular cellular organelles, thereby their proteomes representing mainly the networks associated with the organelles^39^,^40^. To understand the complementary nature of the three proteomes in representing cellular protein networks, we first compared what proportions of the three proteomes included the proteomes of the five major organelles, nucleus, plasma membrane, endoplasmic reticulum (ER)/Golgi apparatus, extracellular region, and cytosol, based on gene ontology cellular components of the proteins identified from the three proteomes (Fig. 2c). Of the three proteomes, the N-glycoproteome most significantly represented the proteins that were localized in plasma membrane (31.0%) or ER/Golgi secretory pathways (22.1%), and secreted to extracellular region (30.2%), consistent to the previous findings^41^.

We then examined the proportions of the three proteomes to represent the proteins in the five types of cellular protein networks in KEGG pathway database^18^: 1) metabolic networks, 2) cellular signaling networks, 3) cell cycle, DNA, and RNA processing networks, 4) protein homeostasis networks, and 5) cellular and organic interaction networks (Supplementary Fig. S3 and Fig. 2d). Of these networks, interestingly, the cellular signaling networks were significantly represented by all the three proteomes (Fig. 2d). Specifically, the N-glycoproteome significantly represented the receptors and their ligands, and the phosphoproteome represented the kinases/phosphatases and the downstream transcription factors (TFs) in the signaling networks (Fig. 2e). In contrast, the global proteome included uniformly all the above groups of the signaling molecules. These data suggest that the three proteomes obtained by the SEPG method provide complementary coverages of cellular protein networks.

### Cellular protein networks altered in gastric cancers

**Figure 3:**
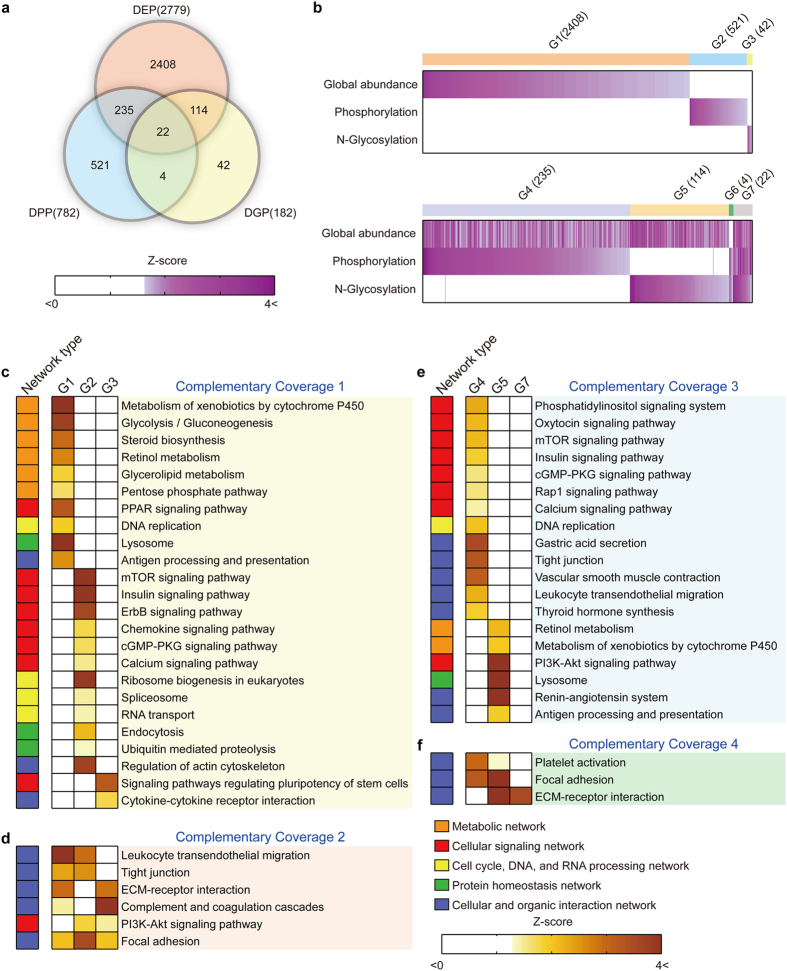
Complementary nature of the three proteomes altered in gastric cancers. (**a**) Relationships among the sets of the altered proteins in gastric cancers (DEPs, DPPs, and DGPs). (**b**) Seven groups of the altered proteins (G1-7) that were further categorized into two classes representing the altered proteins in single types of the three proteomes (G1-3) and more than one type of the three proteomes (G4-7), respectively. (**c–f**) Cellular protein networks in KEGG pathway database associated with Complementary Coverages 1–4 (see text for definition). The color in the heat maps represents the significance measures, *Z* scores defined as –N^−1^(*P*) where N^−1^ is the inverse Gaussian function and *P* is the enrichment *P*-values obtained from ConsensusPathDB software. Color bar, gradients of the *Z* scores. The group to which each cellular protein network belonged was also indicated next to the heat maps (see legend for the network groups).

To understand the proteomes altered in gastric cancers, we first quantified protein abundance changes (log_2_-fold-changes) between tumor and adjacent normal tissues using the iTRAQ data obtained from the global proteome as previously described (**Methods**). From the three tissue pairs, we identified a total of 2,779 differentially expressed proteins (DEPs) between tumor and adjacent normal tissues with P < 0.05 (Supplementary Table S8a and Fig. 3a; Methods). From the phosphoproteome and N-glycoproteome data, we further identified 1,783 differentially phosphorylated peptides [782 differentially phosphorylated proteins (DPPs)], and 521 differentially N-glycosylated peptides [182 differentially glycosylated proteins (DGPs)] with P < 0.05 (Supplementary Table S8b, c and Fig. 3a; Methods). In total, 3,346 proteins were altered in abundances, phosphorylation, or N-glycosylation (Fig. 3a).

The complementary nature of global proteome (DEPs), phosphoproteome (DPPs), and N-glycoproteome (DGPs) can provide the increase in both ‘depth of detection’ and ‘diversity of information’ afforded by the multi-dimensional proteome analyses. To explore the complementary characteristics of the 3,346 proteins in representing cellular networks, we categorized them into seven groups (Groups 1–7) based on their alteration patterns in abundances, phosphorylation, and N-glycosylation (Fig. 3b). Of them, Groups 1–3 showed alterations uniquely in each type of the data. The non-overlapping nature of Groups 1–3 across individual data types provides the increased coverage of the cellular protein networks through the increased depth of detection. To examine this, we identified KEGG pathways represented distinctively by Groups 1–3 (Fig. 3c; Complementary Coverage 1). Group 1 (DEPs) uniquely represented alterations in metabolic networks (glycolysis/gluconeogenesis, pentose phosphate pathway, and glycerolipid/steroid/retinol metabolism). Group 2 (DPPs) uniquely represented alterations in signaling networks (chemokine, calcium, insulin, Erbb, and mTOR signaling pathways). Moreover, Group 3 (DGPs) represented uniquely alterations in cellular and organic interaction networks (cytokine-receptor interaction). These data showed that the complementary coverage 1 (CC1) provided the increased coverage of the altered protein networks in gastric cancers, including metabolic (Group1), signaling (Group 2), and cellular and organic interaction networks (Group 3), by the increased depth of detection.

Alternatively, despite their non-overlapping nature, Groups 1–3 also represented the same protein networks, but different parts of such networks (Fig. 3d; Complementary Coverage 2). Unlike the CC1, the complementary coverage 2 (CC2), which was defined by two of Groups 1–3, provided the increased coverage of cellular protein networks by the increased diversity of information afforded by the multi-dimensional proteome analyses. For example, leukocyte transendothelial migration, a cellular and organic interaction network, was represented by Groups 1 (DEPs) and 2 (DPPs), indicating that the DPPs additionally represented signaling pathways in the network. Similarly, two of the three proteomes collectively represented alterations in cellular and organic interaction networks (focal adhesion, tight junction, and ECM-receptor interaction), and signaling networks (PI3K-AKT signaling pathway).

Moreover, Groups 4–7 showed alterations in more than two types of data. Different types of altered information for the same proteins (e.g., differential expression and phosphorylation for Group 4) can provide complementary states of the proteins in representing activities of the cellular protein networks (Fig. 3e; Complementary Coverage 3). The complementary coverage 3 (CC3) provided the increased coverage of cellular protein networks by the increased diversity of information due to the multiple types of the information in Groups 4–7. To examine this, the pathway enrichment analysis was performed for Groups 4–7. Group 4 represented alterations in signaling networks (mTOR, insulin, cGMP-PKG, and calcium signaling) in terms of protein abundances (DEPs) and signaling activities (DPPs) at the same time. Also, Group 5 represented alterations in metabolic networks (drug and retinol metabolism), signaling networks (PI3K-Akt signaling), and cellular and organic interaction networks (antigen processing and presentation) in terms of protein abundances (DEPs) and secretability (DGPs) at the same time. These data showed that the CC3 provided the increased coverage of the signaling and metabolic networks represented by Groups 4–5 by the multiple types of information in Groups 4–5 (increased diversity of information). Finally, similar to the CC2, more than two of Groups 4–7 also represented the same protein networks, but different parts of such networks (Fig. 3f; Complementary Coverage 4). Due to the nature of Groups 4–7 showing alterations in more than two types of data, the CC4 provided the increased coverage of the altered protein networks by the increased diversity of information, similar to the CC3.

### Complementary coverages of protein networks altered in gastric cancers

To understand complementary coverages of cellular networks altered in gastric cancers at the molecular level, we next constructed the network models delineating the Complementary Coverages 1–4 (CC1-4) using molecular interactions in the KEGG pathway database. Groups 1–3 associated with CC1 and CC2 included larger numbers of the proteins (DEPs, DPPs, and DGPs) altered in gastric cancers, compared to Groups 4–7 associated with CC3 and CC4 (Fig. 3b). Moreover, Groups 4–7 represented only few networks associated with CC4, compared to those with CC3 (Fig. 3e,f). Thus, in this network modeling, we focused on the networks associated with CC1-3.

**Figure 4:**
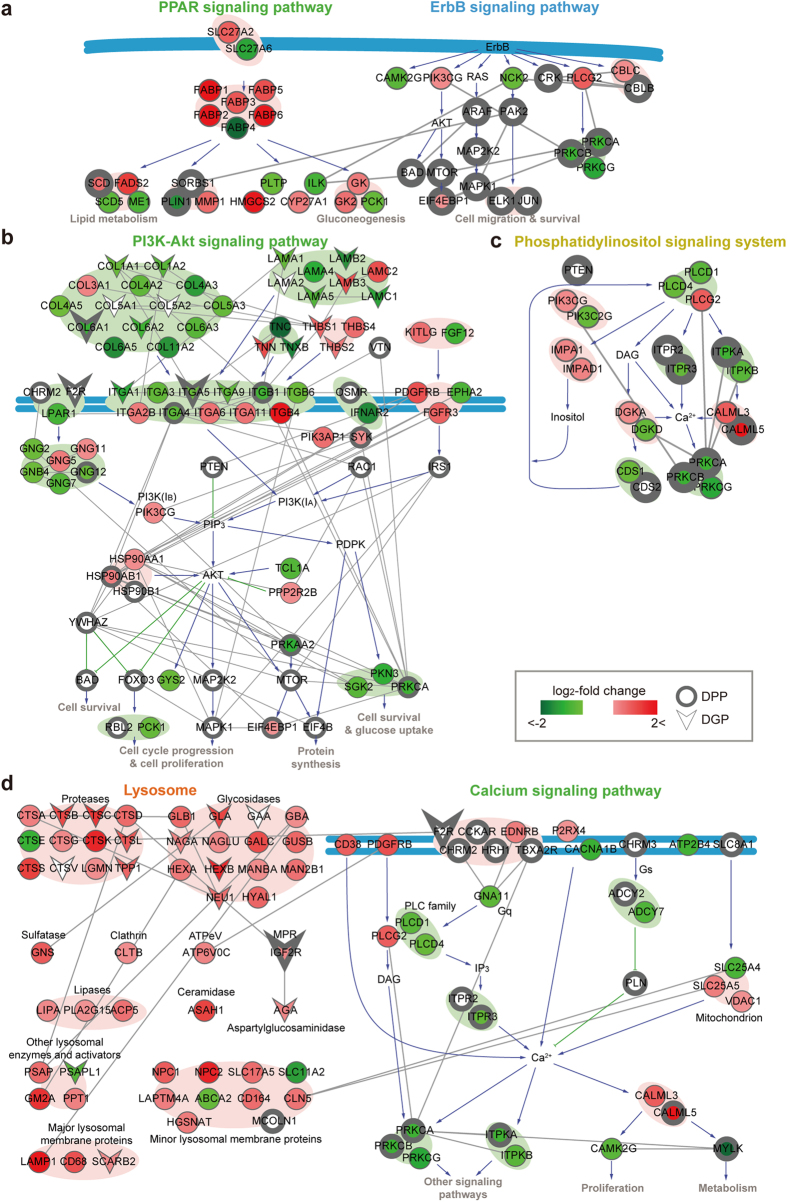
Network models delineating complementary coverages of cellular protein networks provided by the three proteomes. (**a**) PPAR and ErbB signaling networks representing Complementary Coverage 1, which were mainly defined by the DEPs and DPPs, respectively. Colored nodes, nodes with gray boundary, and V-shaped nodes represent the DEPs, DPPs, and DGPs, respectively. The nodes in the network models were arranged based on the structures of the corresponding KEGG networks. The arrows and inhibition symbols represent the activation and repression information, respectively, obtained from the KEGG networks and gray edges represent PPIs among the nodes obtained from the six databases (**Methods**). Plasma membrane was denoted as the blue lines. Backgrounds represent functional modules in the KEGG networks. (**b**) PI3K-AKT signaling network representing Complementary Coverage 2 and 3. (**c**) Phosphatidylinositol signaling system network representing Complementary Coverage 3. (**d**) Cellular networks defined by two types of the complementary coverages (Complementary Coverage 1 and 3): lysosome network defined by Groups 1 and 5 and calcium signaling network defined by Groups 2 and 4.

First, the networks for CC1 were uniquely defined by single types of the proteomes altered in gastric cancers (Fig. 4a). PPAR signaling network (Fig. 4a, left) showed alterations of fatty acid uptake (SLC27A2/6) and transports (FABP1-6) in gastric cancers, leading to dysregulated activation of PPAR pathways, resulting in alteration of PPAR targets involved in lipid homeostasis. These alterations were defined mainly by the DEPs. In contrast, alterations of ErbB signaling network (Fig. 4a, right) were mainly defined by the DPPs including protein kinase C (PRKCA/B) and mitogen activated protein kinase (ARAF, MAP2K2, and MAPK1), and mTOR (MTOR and EIF4EBP1) signaling molecules, as well as their downstream TFs (ELK1 and JUN).

Second, the networks for CC2 were collectively defined by two types of the proteomes. PI3K-AKT signaling network (Fig. 4b) showed alterations of upstream regulators (collagens, laminins, and integrins) and signaling molecules (PTEN, RAC, MAPK, and MTOR) and downstream TFs (FOXO3 and RBL2). Many of these molecules belonged to the DGPs and DPPs, indicating that both phosphoproteome and N-glycoproteome collectively defined the altered activity of PI3K-AKT network in gastric cancers. Interestingly, the DEPs also provided additional information for the altered network in terms of protein abundances, although the enrichment P-value was not significant due to the large number of the DEPs in Group 1.

Third, the networks for CC3 were commonly defined by two types of the proteomes. Phosphatidylinositol signaling system network (Fig. 4c) included inositol trisphosphate receptors and kinases (ITPR2, ITPR3, and ITPKA) and their downstream signaling molecules (CAML5, PRKCA, and PRKCB), which belonged to the DEPs and DPPs, indicating that their abundances and phosphorylation levels both were collectively altered in gastric cancers. Finally, a set of the networks were associated with multiple CCs. Lysosome (Fig. 4d, left) was associated with both CC1 and CC3 (Fig. 3c,e). CC1 was defined by Group 1 (DEPs), while CC3 was by Group 5 (DEPs and DGPs). Similarly, calcium signaling network (Fig. 4d, right) was associated with both CC1 and CC3 (Fig. 3c,e) defined by the DPPs and/or DEPs (Groups 2 and 4). The pathways in the network models were previously reported in association with gastric cancers (Supplementary Table S9), supporting the validity of the network models. Thus, these networks demonstrated the power of complementary coverages provided by the three proteomes in understanding altered activities of the protein networks in gastric cancers.

### Effective prioritization of key network regulators by complementary coverages

The improved network coverage by multi-dimensional proteomes can provide additional insights into functions of the networks. To test this, we investigated whether the improved coverage enabled effective prioritization of key regulators in the networks. First, a hub-like molecule with a large number of interactors critically affects functions of the networks^42^. Thus, we examined whether the improved coverages affected identification of hub-like molecules. To this end, we identified 154, 232, 160, and 233 hub-like molecules for the four sets of altered molecules (DEPs, DEP + DPPs, DEP + DGPs, and DEP + DPP + DGPs), respectively, based on the protein-protein interactome data obtained from the six databases as described in **Methods**.

**Figure 5:**
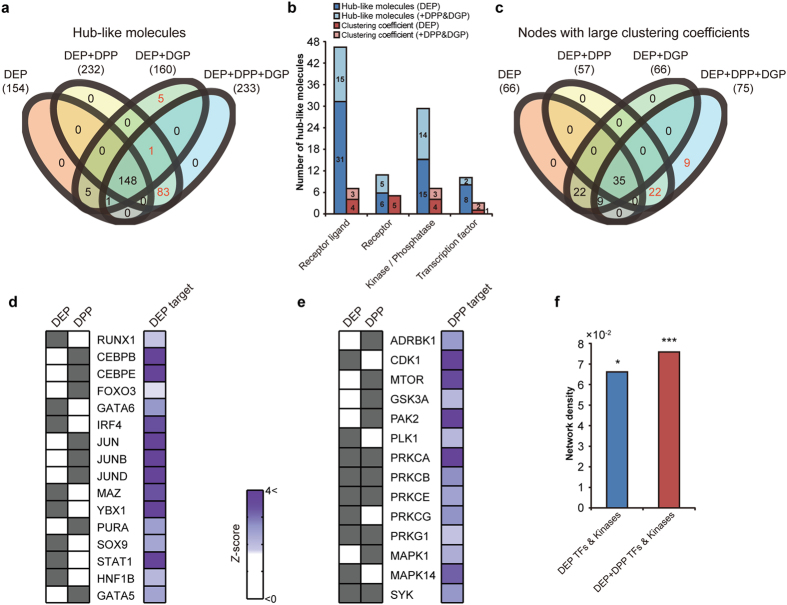
Effective identification and prioritization of key network regulators by the complementary multi-dimensional proteomes. (**a**) Relationships of hub-like molecules identified for the four sets of the altered proteins in gastric cancers: DEPs, DEP + DPPs, DEP + DGPs, and DEP + DPP + DGPs. Total numbers of hub-like molecules identified for the four sets were denoted in parenthesis. Red numbers indicate additional hub-like molecules identified by including DPPs or DGPs. (**b**) Numbers of hub-like molecules involved in the indicated four groups of signaling molecules. Colored stacked bar graphs showed incremental identifications of hub-like molecules and nodes with large clustering coefficients by the indicated multi-dimensional proteomes (see color legend). (**c**) Relationships of nodes with large clustering coefficients identified for the four sets of the altered proteins. (**d-e**) Key TFs (**d**) and kinases (**e**) identified using the DEPs and DPPs. *Z* scores represent the significance of the TFs and kinases having their downstream targets. Color bar, gradients of the *Z* scores. (**f**) Increased density of the networks describing interactions among the key TFs and kinases by the multi-dimensional proteomes. *P < 0.05; ***P < 0.001.

Comparison of hub-like molecules for the four sets showed that DEP + DPPs and DEP + DGPs identified additionally 84 and 6 hub-like molecules, respectively (Fig. 5a), compared to the DEPs, and DEP + DPP + DGPs identified additionally 89 hub-like molecules, indicating that the multi-dimensional proteomes enabled identification of hub-like molecules unidentifiable using single types of the proteomes. Interestingly, of the hub-like molecules, 148 were identified for all the four sets, suggesting their reliability as hub-like molecules. To examine how the extended list of hub-like molecules contributed to interpretation of cellular networks, we analyzed the numbers of the hub-like molecules in the four groups of signaling molecules (ligands, receptors, kinases/phosphatases, and TFs) and found that DPPs and DGPs provided additional hub-like molecules of kinases/phosphatases and receptor ligands, respectively, thereby enhancing understanding of their functions in gastric cancer-related networks (Fig. 5b).

Next, clustering coefficients for nodes represent how densely interactors of the nodes are connected. The nodes with large clustering coefficients (NLCCs) can be highly influential in functions of the networks through dense connections with their interactors. Thus, we examined whether the improved coverage affected identification of NLCCs. We first identified 66, 57, 66, and 75 NLCCs for DEPs, DEP + DPPs, DEP + DGPs, and DEP + DPP + DGPs, respectively (**Methods**). Similarly, DEP + DPPs and DEP + DPP + DGPs identified additional 22 and 31 NLCCs, respectively (Fig. 5c), compared to the DEPs, which provided additional NLCCs of kinases/phosphatases and receptor ligands, as in the case of the hub-like molecules (Fig. 5b). Of the NLCCs, 35 were identified for all the four sets of altered molecules, thereby enabling effective prioritization of NLCCs in gastric cancer-related networks.

The TFs and kinases having significant numbers of altered proteins as downstream targets can be considered as key regulators underlying alterations in the measured proteomes. To identify these key TFs and kinases, we performed the enrichment analyses of TFs and kinases for the DEPs and DPPs based on the 304,217 protein-DNA and 5,563 kinase-substrate interaction data, respectively, obtained from the seven databases and the three databases, as described in **Methods**, and then selected the key TFs and kinases (P < 0.05) whose expression or phosphorylation levels were altered in gastric cancers. Only using the DEPs, we identified eight key TFs (RUNX1, GATA6, IRF4, MAZ, YBX1, SOX9, STAT1, and HNF1B), but using DEP + DPPs, we identified eight key TFs additionally (CEBPB/E, FOXO3, JUN, JUNB/D, PURA, and GATA5) (Fig. 5d). Similarly, using DEP + DPPs, the kinase enrichment analysis identified five key kinases (ADRBK1, MTOR, GSK3A, PAK2, and MAPK1) additionally to nine key kinases identified from the DEPs only (Fig. 5e). The increased numbers of key kinases and TFs led to improved coverage of cellular networks represented by them, thereby facilitating interpretation of the key protein networks altered in gastric cancers (Fig. 5f).

## Discussion

How much added value the multi-dimensional proteomic analyses can provide in understanding cellular networks is unclear, despite significant amounts of resources required for the multi-dimensional analyses. Here, we first proposed the SEPG method for effective multi-dimensional proteomic analyses. In the SEPG, mRP fractionation provided complex peptide mixtures with uniform and wide separation space, and the use of TEAB as buffer solvents provided compatibility with iTRAQ samples and high volatility, leading to no desalting steps during the entire procedure, and also low detrimental effects on separation columns. Thus, the SEPG resulted in large proteome coverages, alleviating the undersampling issues. Furthermore, the FASP digestion methods used in the SEPG provided a wide range of the proteome that includes nuclear, membranous, and secreted extracellular proteins. The larger and wider proteomes demonstrated the utility of the SEPG as an effective approach for multi-dimensional proteomic analyses. Importantly, the proposed SEPG, compared to previous methods, is unique in that it was successfully applied to human patient tissues for the multi-dimensional analyses.

In this study, we performed multi-dimensional proteomic profiling on the pairs of tumor and adjacent normal tissues collected from three different patients (n = 3). The reproducibility of proteomic analysis is essential to draw reliable conclusions on the complementary coverages of cellular networks altered in gastric cancers. For each patient, we generated two independent replicates from the tumor and adjacent normal tissue masses, respectively, and then labeled the resulting four independent peptide samples using iTRAQ (Supplementary Methods). To assess the reproducibility in our proteomic analysis, we performed two analyses that have been used to assess the reproducibility of MS-based proteomic analysis. First, we evaluated the similarity of the two replicates generated for adjacent normal or tumor tissue from each patient in intensities of identified peptides (Supplementary Fig. S4), which provides the assessment of the reproducibility in the early steps of sample preparation (lysis, protein isolation and digestion, and labeling). Large correlation coefficients (≥0.95) in the three types of proteomic data suggest high reproducibility in the early steps of sample preparation. Second, we also evaluated the similarities of identified peptides (ID similarity) and their intensities (Intensity similarity) in the data obtained from the three patients as previously reported for the three types of proteomic data (Supplementary Fig. S5), which provides the assessment of the reproducibility in the late steps of sample preparation (fractionation and enrichment) and LC-MS/MS analysis for three different patients. Overall similarity scores were found to be sufficiently high (average overall similarity scores = 0.77, 0.66, and 0.71 for global protein expression, phosphopeptide, and N-glycopeptide data), suggesting the consistency in the late steps of sample preparation and LC-MS/MS analysis for three different patients. Collectively, all these data suggest high quality of proteomic analyses and resulting data given the variability in the sample, both the early and late steps of sample preparation, as well as LC-MS/MS and data analyses.

In this study, we pooled the three sets of proteins (Global) and differentially expressed proteins (DEPs) identified from global protein expression data generated from three gastric cancer patients into ‘Global set’ and ‘DEP set’, respectively. Similarly, proteins that have phosphopeptides (Phospho), differentially phosphorylated peptides (DPPs), N-glycopeptides, and differentially glycosylated peptides (DGPs) identified from the three patients were pooled into ‘Phospho set’, ‘DPP set’, ‘N-glyco set’, and ‘DGP set’, respectively. These pooled sets were used to map the detected proteins into cellular networks (Global, Phospho, and N-glyco sets; Fig. 2), to analyze complementary coverages of cellular networks provided by the multi-dimensional data (DEP, DPP, and DGP sets; Fig. 3), and to reconstruct the network models for the complementary coverages (Fig. 4). However, with the pooled sets, it is not clear whether the results from the individual analyses mentioned above were consistent in all three patients. To identify patient-specific information (e.g., subtypes of gastric cancers and proteomic signatures defining the subtypes), the multi-dimensional proteomic analysis should be performed for a large size of samples (e.g., hundreds of samples), as demonstrated in the TCGA genomic analyses. However, the main goal in this study is to demonstrate that the multi-dimensional proteomic analysis provides complementary coverages of cellular networks, rather than to identify the subtypes of patients. Nonetheless, it is important to draw reliable conclusions from the individual analyses, which hold in all three patients. To examine this aspect, we analyzed whether the multi-dimensional data generated from three different patients consistently contributed to the outputs from the individual analyses. To this end, we identified the sets individually (individual sets) from the three patients (e.g., Global sets 1–3 for patients 1–3, respectively) and then analyzed how many of the proteins in the individual sets contributed to the analysis outputs obtained from the pooled sets. For example, we showed that the three proteomes increased cumulatively the coverage of the cellular proteome, as shown in Fig. 2b. Consistently, the increase was observed consistently in all the three patients (Supplementary Fig. S6a), indicating that the individual sets obtained from the three patients consistently contributed to the increased coverage shown in Fig. 2b. Also, the individual sets consistently contributed to the following conclusions obtained from the pooled sets: 1) preferential enrichment of N-glycosylated proteins in plasma membrane, ER/Golgi secretory pathways, and extracellular region (Supplementary Fig. S6b) and also in receptor ligands and receptors of cellular signaling networks (Supplementary Fig. S6c); 2) preferential enrichment of phosphorylated proteins in kinases/phosphatases of cellular signaling networks (Supplementary Fig. S6c); 3) complementary coverages (CC1-4) of cellular networks defined by the multi-dimensional data shown in Figs 3c–f (Supplementary Fig. S6d-g); and network models for the complementary coverages shown in Fig. 4 (Supplementary Fig. S6h-k). All these data suggest that the three patients consistently contributed to the outputs resulted from the individual analyses, thus supporting the validity of the conclusions drawn from the pooled sets.

The integrated analyses of the three proteomes generated by the SEPG further revealed the power of multi-dimensional proteomic analyses in understanding of disease-related networks. Phosphoproteome captured predominantly signaling proteins (kinases/phosphatases and TFs) in disease-related signaling networks, while N-glycoproteome captured the proteins localized in plasma membrane, ER/Golgi apparatus, and extracellular regions (receptor ligands and receptors). Global proteome also provided protein abundances uniformly across the five groups of disease-related networks (Supplementary Fig. S3 and Fig. 2d). Finally, the integrated analysis of altered proteins under diseased conditions enabled effective identification and prioritization of key regulators (hub-like molecules, NLCCs, or key TFs/kinases) in disease-related networks. Therefore, our integrated analyses demonstrated that the three proteomes generated by the SEPG systematically improved the coverages of cellular networks, thereby facilitating functional interpretation of disease-related networks.

In summary, our multi-dimensional proteomic approach, including the SEPG and the integrated analyses of the three proteomes, provided new knowledge regarding alterations of disease-related networks in terms of CC1-4 of cellular networks and also key network regulators. This knowledge can be used as a comprehensive basis to understand functions of disease-related networks. Furthermore, our approach can be applied to other diseases in which multi-dimensional proteomic analyses are needed because single types of the proteomes (e.g. global proteomes) have failed to understand core disease-related networks and/or to identify key regulators of the disease-related networks.

## Additional Information

**How to cite this article**: Park, J.-M. *et al.* Integrated analysis of global proteome, phosphoproteome, and glycoproteome enables complementary interpretation of disease-related protein networks. *Sci. Rep.* **5**, 18189; doi: 10.1038/srep18189 (2015).

## Supplementary Material

Supplementary Information

Supplementary Table S2a

Supplementary Table S2b

Supplementary Table S2c

Supplementary Table S7a

Supplementary Table S7b

Supplementary Table S7c

Supplementary Table S8a

Supplementary Table S8b

Supplementary Table S8c
