# Supplementary material for: Integrated analysis of global proteome, phosphoproteome, and glycoproteome enables complementary interpretation of disease-related protein networks
Source: Sci Rep. 2015 Dec 11;5:18189. doi: 10.1038/srep18189 (PMC4676070; doi:10.1038/srep18189)
Supplement: Supplementary Information [file srep18189-s1.doc]

**SUPPLEMENTARY INFORMATION** for

**Integrated analysis of global proteome, phosphoproteome, and glycoproteome enables complementary interpretation of disease-related protein networks**

Jong-Moon Park^1,†^, Ji-Hwan Park^2,†^, Dong-Gi Mun^3,†^, Jingi Bae^3,†^, Jae Hun Jung^4^, Seung-hoon Back^3^, Hangyeore Lee^3^, Hokeun Kim^3^, Hee-Jung Jung^6^, Hark Kyun Kim^5^, Hookeun Lee^1^, Kwang Pyo Kim^4^,*, Daehee Hwang^2,6^,*, Sang-Won Lee^3^,*

^1^Department of Pharmaceutics, College of Pharmacy, Gachon University, Incheon 406-799, Republic of Korea; ^2^Department of Chemical Engineering, POSTECH, Pohang 790-784, Republic of Korea; ^3^Department of Chemistry, Research Institute for Natural Sciences, Korea University, Seoul 136-701, Republic of Korea; ^4^Department of Applied Chemistry, College of Applied Sciences, Kyung Hee University, Yong-in 446-701, Republic of Korea; ^5^National Cancer Center, Goyang 410-769, Republic of Korea; and ^6^Department of New Biology and Center for Plant Aging Research, Institute for Basic Science, DGIST, Daegu 711-873, Republic of Korea.

^†^These authors equally contributed to this work.

*Corresponding authors:

Sang-Won Lee, Ph.D.,

Department of Chemistry, Research Institute for Natural Sciences, Korea University, Seoul 136-701, Republic of Korea;

Phone: 82-2-3290-3603; Fax: 82-2-3290-3121; and E-mail: sw_lee@korea.ac.kr;

Daehee Hwang, Ph.D.,

Department of New Biology and Center for Plant Aging Research, Institute for Basic Science, DGIST, Daegu 711-873, Republic of Korea;

Phone: 82-53-785-1840; Fax: 82-53-785-1809; E-mail: dhwang@dgist.ac.kr;

Kwang Pyo Kim, Ph.D.,

Department of Applied Chemistry, College of Applied Sciences, Kyung Hee University, Yong-in 446-701, Republic of Korea;

Phone: 82-31-201-3868; Fax: 82-31-201-2340; E-mail: [kimkp@khu.ac.kr](mailto:kimkp@khu.ac.kr);

# SUPPLEMENTARY FIGURES


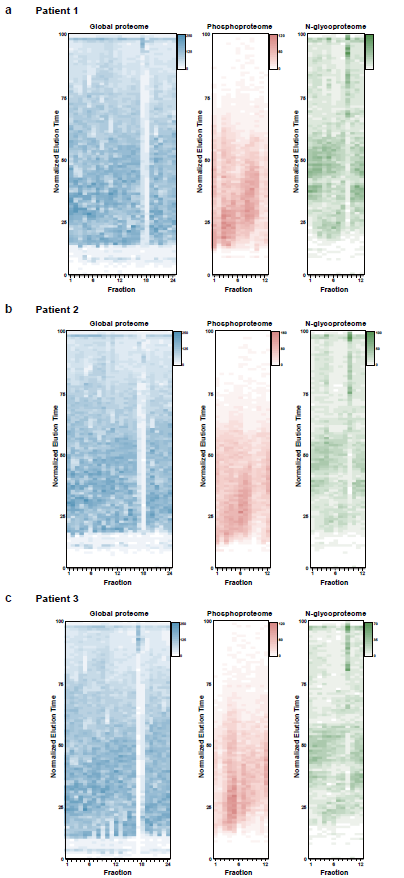


Supplementary Fig S1 **Heat map of the numbers of identified peptide** of patient 1 (a), patient 2 (b) and patient 3 (c) for global proteome, phosphoproteome and N-glycoproteome, respectively.


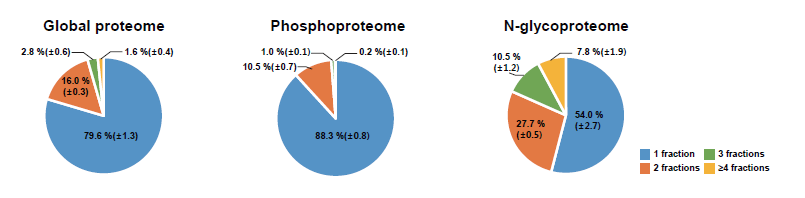


Supplementary Fig S2. **Detection frequencies of peptides over 24 and 12 factions in global proteome and phosphoproteome/N-glycoproteome**. Frequencies were categorized into 1 to 3 and more than 4 as shown in the legend.

**
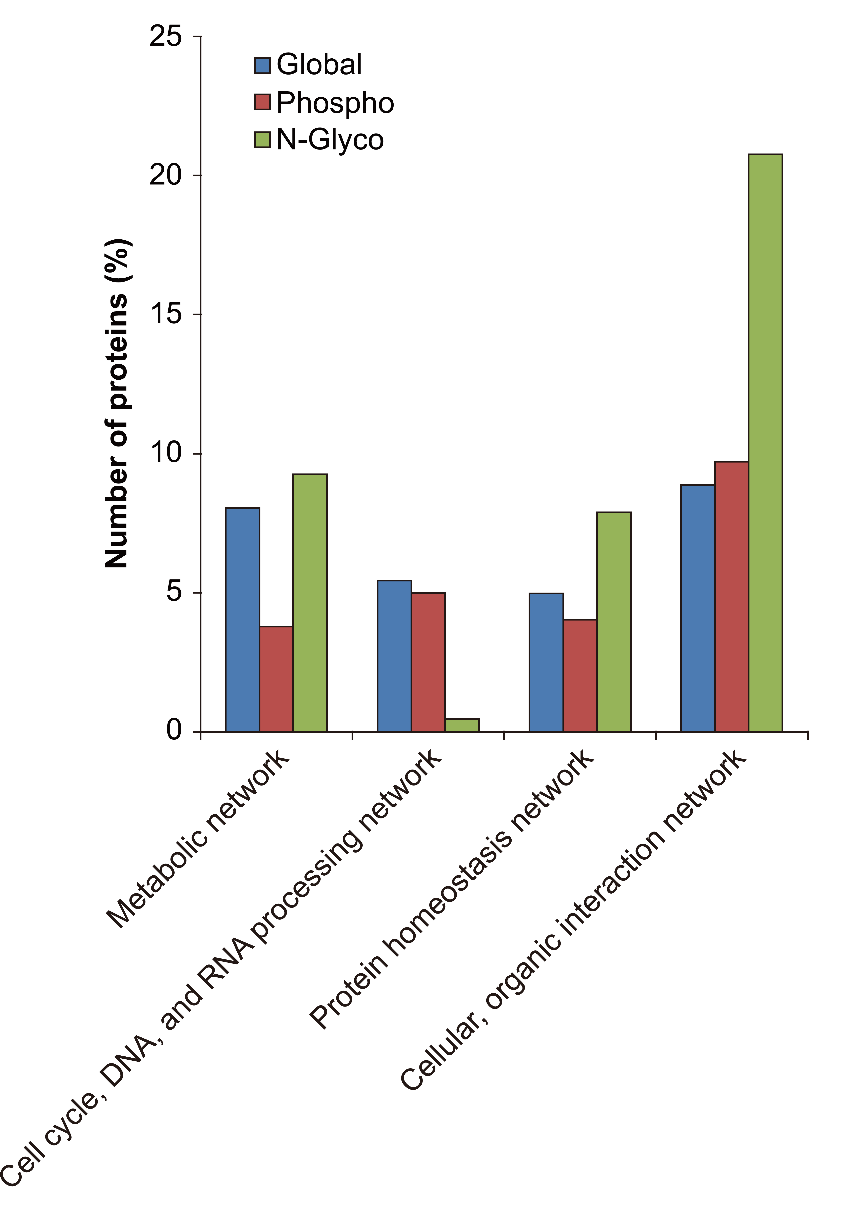
**

Supplementary Fig S3. **The three proteomes in the four types of cellular protein networks.** The five types of cellular protein networks were categorized based on KEGG pathway database. For the four of the five types of cellular protein networks, percentages of proteins measured by the three proteomes were shown in the figure.


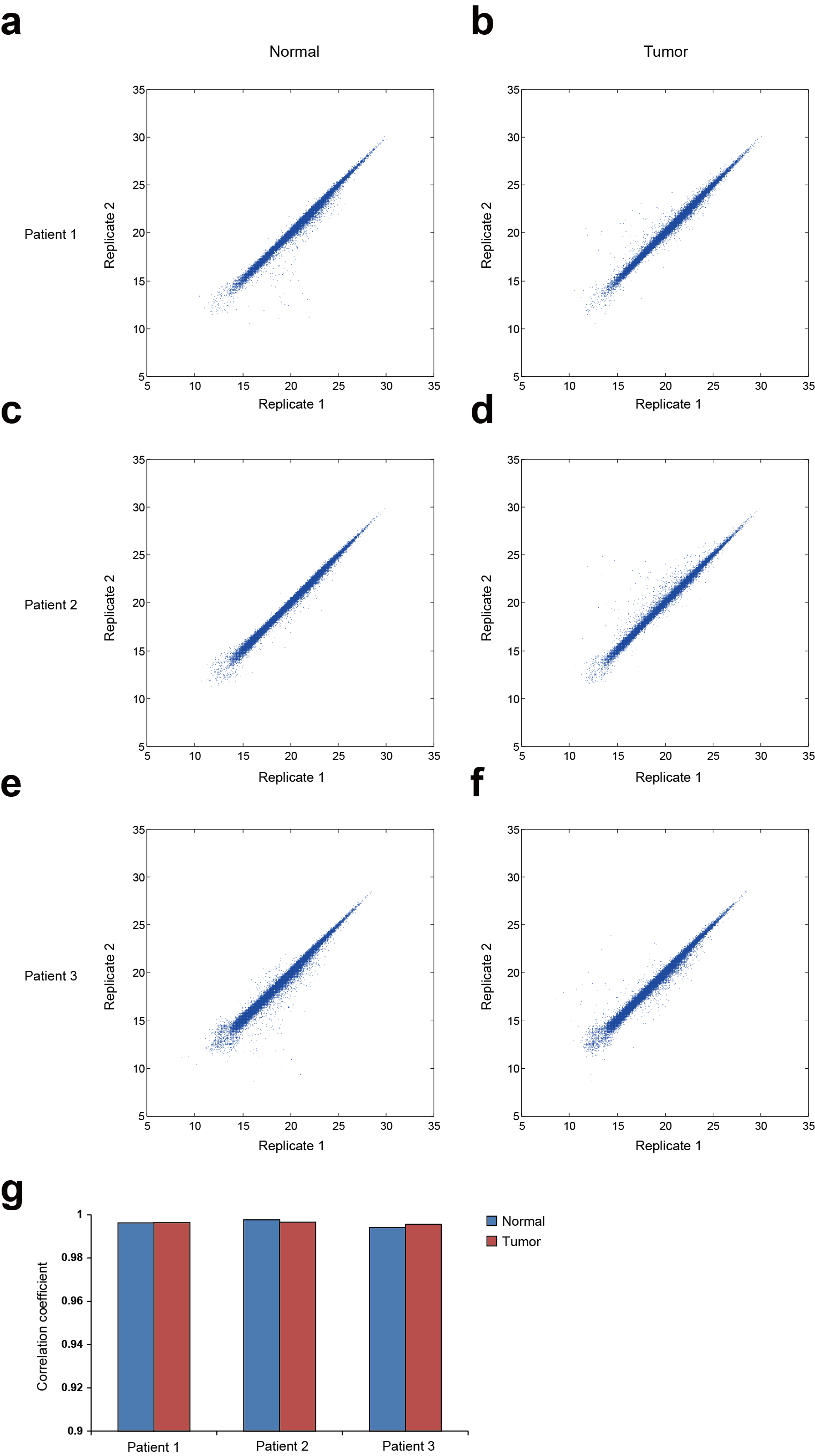

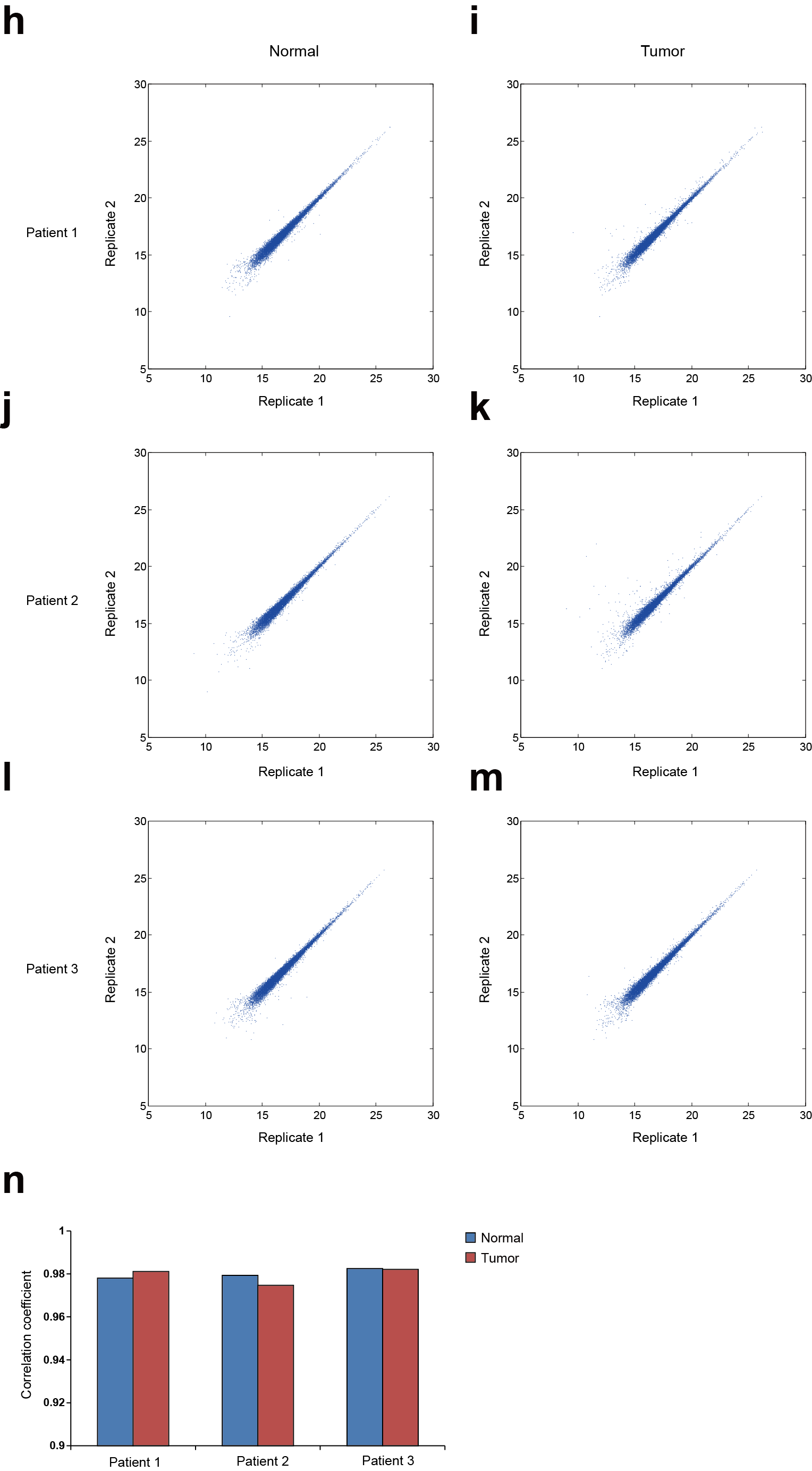

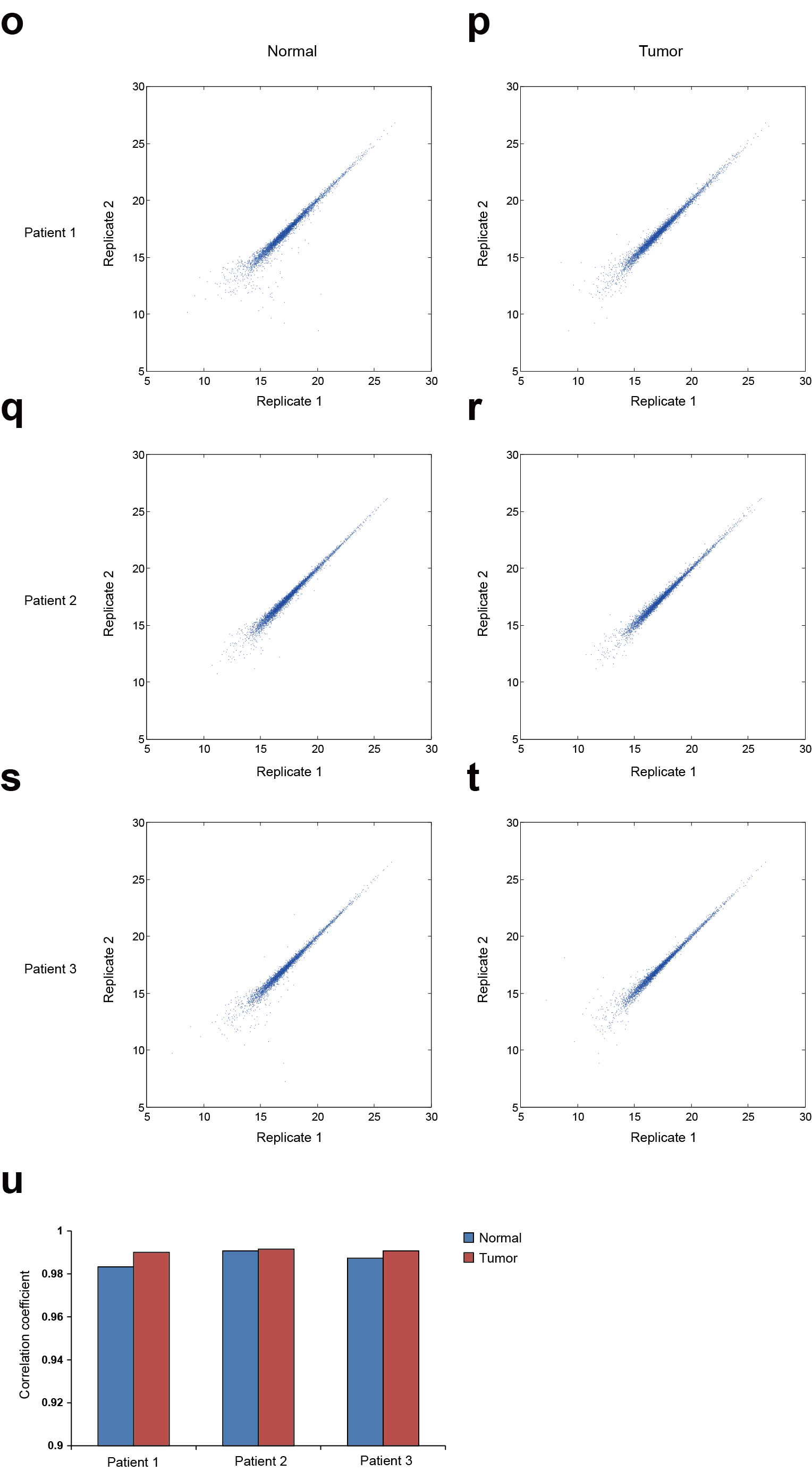


Supplementary Fig S4. **Reproducibility between two technical replicates in three types of proteomic data.**

**(a-f)** Scatter plots of the two technical replicates for global protein expression data generated from normal (left columns) and tumor (right columns) tissues collected from patients 1-3 (rows 1-3, respectively). **(g)** Spearman correlation coefficients for the global protein expression data in the scatter plots in patients 1-3. **(h-m)** Scatter plots of the two technical replicates for phosphorylated peptide data generated from normal (left columns) and tumor (right columns) tissues collected from patients 1-3 (rows 1-3, respectively). (**n**) Spearman correlation coefficients for the phosphorylated peptide data in the scatter plots in patients 1-3. **(o-t)** Scatter plots of the two technical replicates for N-glycosylated peptide data generated from normal (left columns) and tumor (right columns) tissues collected from patients 1-3 (rows 1-3, respectively). (**u**) Spearman correlation coefficients for the N-glycosylated peptide data in the scatter plots in patients 1-3.


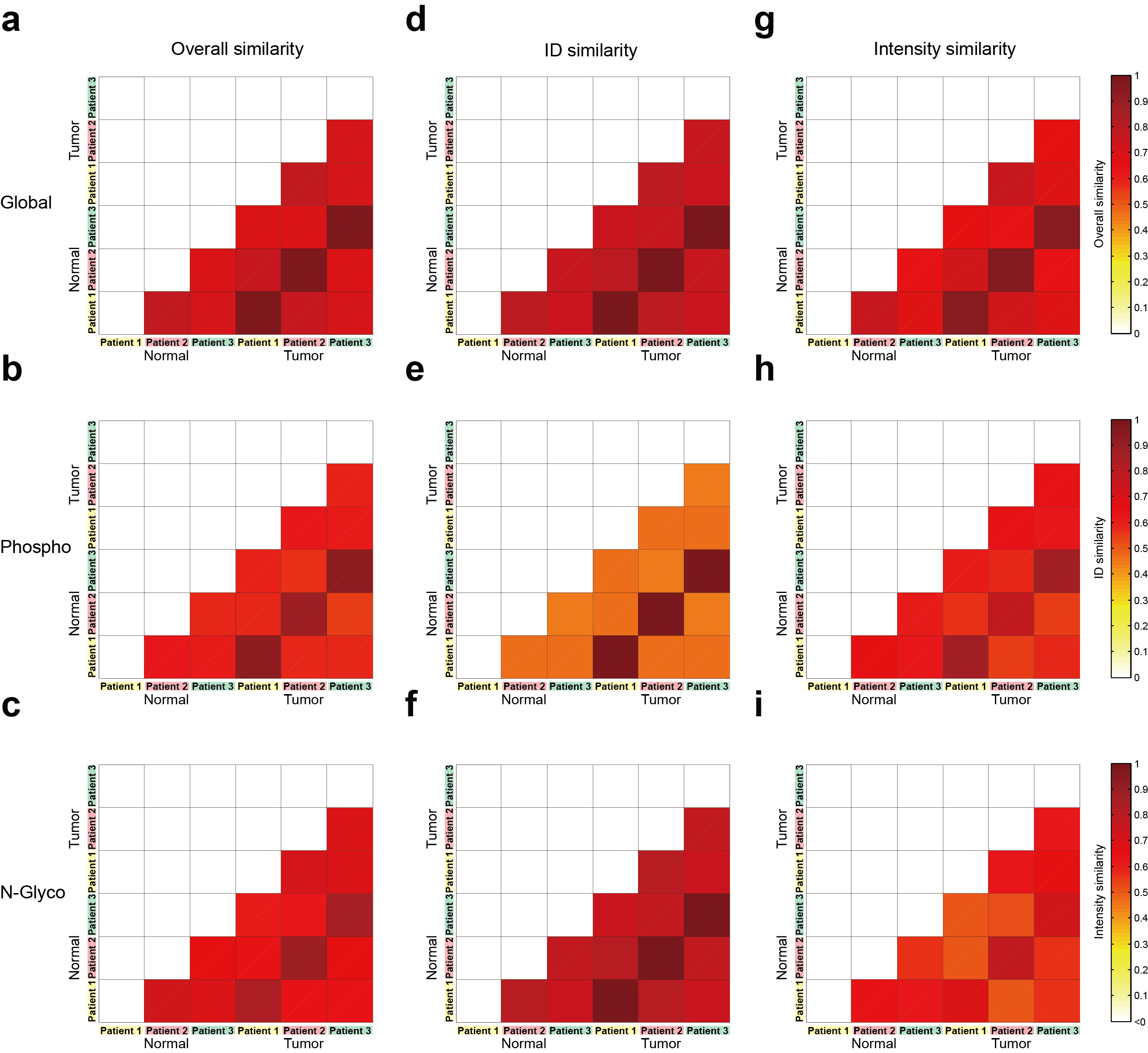


Supplementary Fig S5. **Similarities between the proteomic data obtained from three different patients.**

**(a-c)** Pair-wise overall similarities (geometric mean of ID and intensity similarities) for global protein expression **(a)**, phosphorylated peptide **(b)**, and N-glycosylated peptide data **(c)** for the six samples (3 normal and 3 tumor samples) from the three patients. **(d-f)** Pair-wise ID similarities for global protein expression **(d)**, phosphorylated peptide **(e)**, and N-glycosylated peptide data **(f)** for the six samples (3 normal and 3 tumor samples) from the three patients. **(g-h)** Pair-wise intensity similarities (Spearman correlation coefficient) for global protein expression **(g)**, phosphorylated peptide **(h)**, and N-glycosylated peptide data **(i)** for the six samples (3 normal and 3 tumor samples) from the three patients. The intensity similarity between sample *i* and *j* was calculated using the mean values of peptide intensities in the two technical replicates in each sample. The color bars indicate the gradients of overall, ID and intensity similarity values.


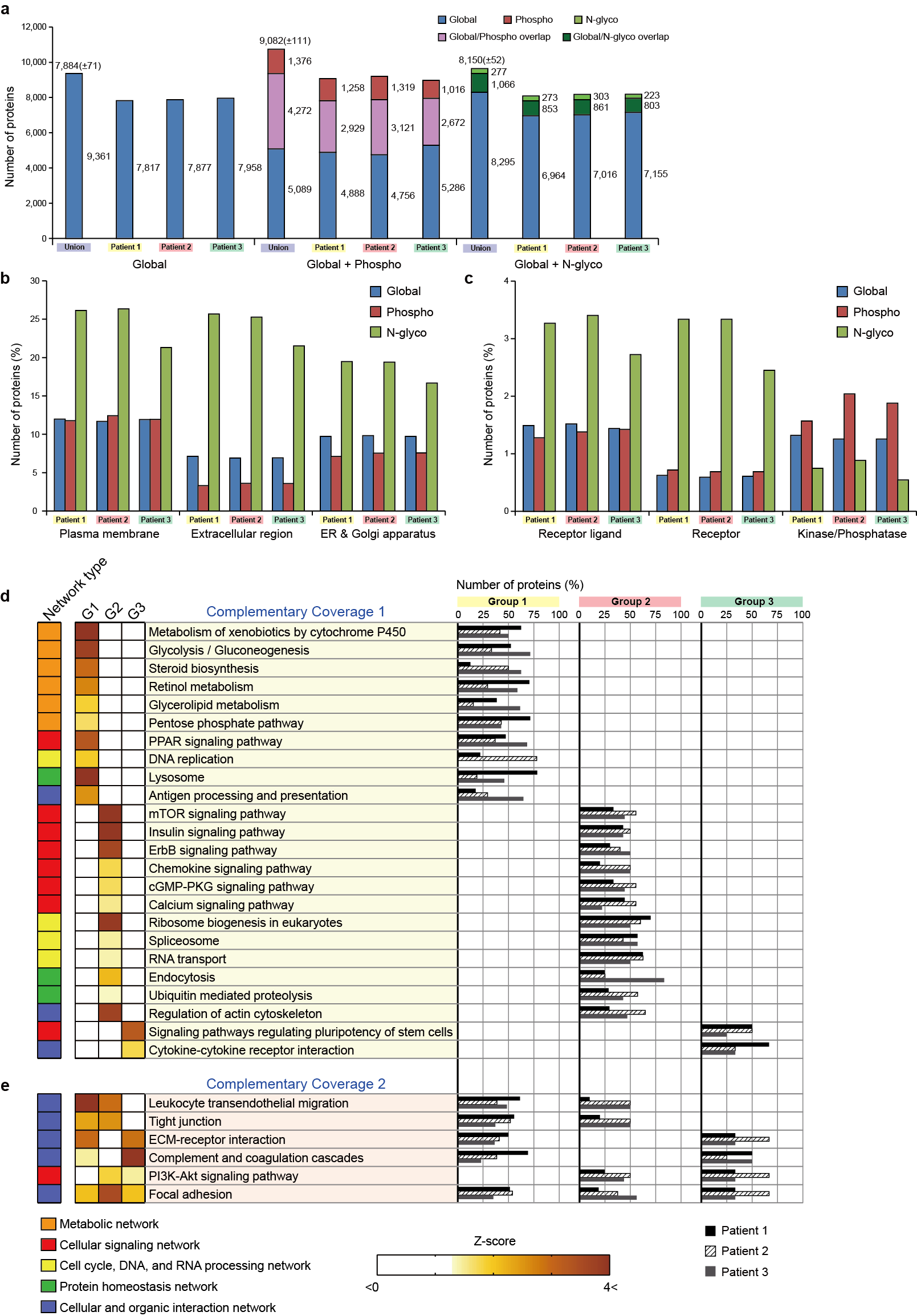

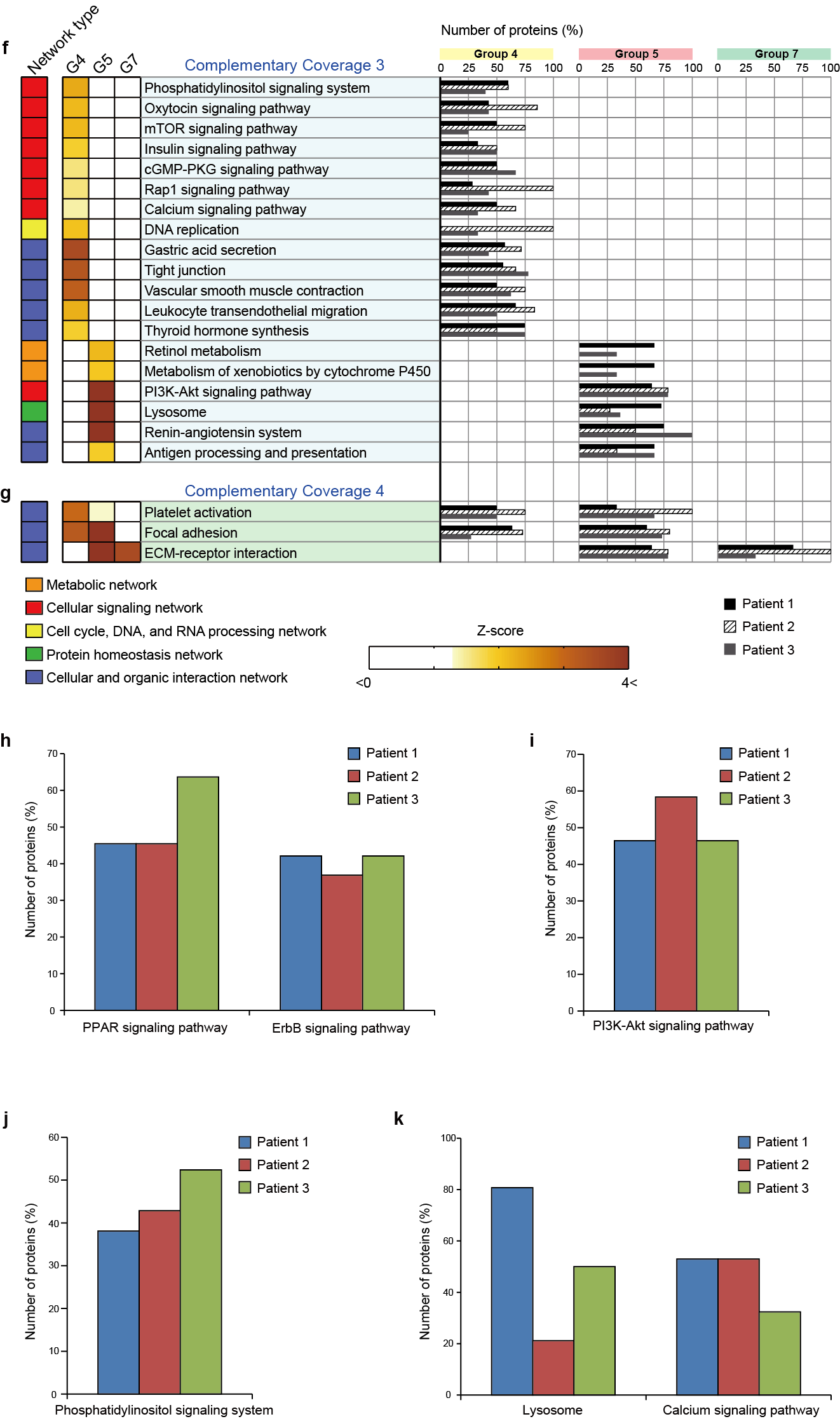


Supplementary Fig S6. **Patient-wise properties of the three proteomes.**

**(a)** Numbers of protein coding genes measured by the three proteomes in three patients. Colored stacked bar graphs showed incremental identifications of the genes by the indicated multi-dimensional proteomes in individual patients. **(b)** Percentages of proteins localized in the indicated major organelles, based on their gene ontology cellular components. The percentages were calculated for the three proteomes in individual patients. **(c)** Percentages of proteins involved in the cellular signaling networks belonging to the indicated groups of signaling molecules. The percentages were calculated for the three proteomes in individual patients. **(d-g)** Cellular protein networks in KEGG pathway database associated with Complementary Coverages 1-4. The color in the heat maps represents the significance measures, *Z* scores defined as –*N*^-1^(*P*) where *N*^-1^ is the inverse Gaussian function and *P* is the enrichment *P*-values obtained from ConsensusPathDB software. Color bar, gradients of the *Z* scores. The group to which each cellular protein network belonged was also indicated next to the heat maps (see legend for the network groups). The bar graph for each protein network shows relative fractions of proteins involved in the protein network measured in individual patients. **(h-k)** Percentages of proteins involved in the following network models for the four complementary coverages shown in Figure 4: PPAR signaling pathway and ErbB signaling pathway (CC1; **h**), PI3K-Akt signaling pathway (CC2 and CC3; **i**), phosphatidylinositol signaling system (CC3; **j**), and lysosome and calcium signaling pathway (CC1 and CC3; **k**). The percentages were calculated separately in individual patients.

# SUPPLEMENTARY TABLES

Supplementary Table S1. **Sample information of three gastric cancer patients.**

Gender, age and tumor stage of three patients are indicated. The average protein extraction yield was measured to be ca. 10 % of wet tissue weight. After FASP digestion, ca. 6 w/w % peptides were obtained from wet tissue.

|  | **Patient 1 Normal** | **Patient 1 Cancer** | **Patient 2 Normal** | **Patient 2 Cancer** | **Patient 3 Normal** | **Patient 3 Cancer** |
| --- | --- | --- | --- | --- | --- | --- |
| **Gender** | Male | | Male | | Female | |
| **Age (year)** | 40 | | 44 | | 45 | |
| **Tumor stage** | IIB | | IIB | | IV | |
| **Tissue (mg)** | 85.00 | 80.00 | 84.00 | 75.00 | 65.10 | 66.10 |
| **Protein (mg)** | 7.12 | 6.67 | 7.36 | 7.60 | 7.14 | 8.18 |
| **Protein extraction yield from tissue (%)** | 8.38 | 8.34 | 8.76 | 10.13 | 10.96 | 12.37 |
| **Protein used for FASP digestion (mg)** | 7.00 | 6.67 | 7.00 | 7.00 | 3.50 | 3.50 |
| **Digested peptide (mg)** | 4.42 | 3.83 | 4.54 | 4.20 | 1.98 | 1.99 |
| **Peptide yield from tissue (%)** | 5.29 | 4.79 | 5.67 | 6.08 | 6.21 | 7.05 |

Supplementary Table S3. **The five types of cellular protein networks.**

The cellular protein networks were categorized based on KEGG pathway database to following five groups: 1) metabolic networks, 2) cellular signaling networks, 3) cell cycle, DNA, and RNA processing networks, 4) protein homeostasis networks, and 5) cellular and organic interaction networks. For each group, the KEGG pathway terms and their categories in KEGG pathway database were shown in the table.

| **Network types** | **Categories in KEGG pathway DB** | **KEGG pathways** |
| --- | --- | --- |
| Group 1 | 1.1 Carbohydrate metabolism | Glycolysis / Gluconeogenesis |
|  |  | Citrate cycle (TCA cycle) |
|  |  | Pentose phosphate pathway |
|  |  | Pentose and glucuronate interconversions |
|  |  | Fructose and mannose metabolism |
|  |  | Galactose metabolism |
|  |  | Ascorbate and aldarate metabolism |
|  |  | Starch and sucrose metabolism |
|  |  | Amino sugar and nucleotide sugar metabolism |
|  |  | Pyruvate metabolism |
|  |  | Glyoxylate and dicarboxylate metabolism |
|  |  | Propanoate metabolism |
|  |  | Butanoate metabolism |
|  |  | Inositol phosphate metabolism |
|  | 1.2 Energy metabolism | Oxidative phosphorylation |
|  |  | Nitrogen metabolism |
|  |  | Sulfur metabolism |
|  | 1.3 Lipid metabolism | Fatty acid biosynthesis |
|  |  | Fatty acid elongation |
|  |  | Fatty acid degradation |
|  |  | Synthesis and degradation of ketone bodies |
|  |  | Steroid biosynthesis |
|  |  | Primary bile acid biosynthesis |
|  |  | Steroid hormone biosynthesis |
|  |  | Glycerolipid metabolism |
|  |  | Glycerophospholipid metabolism |
|  |  | Ether lipid metabolism |
|  |  | Sphingolipid metabolism |
|  |  | Arachidonic acid metabolism |
|  |  | Linoleic acid metabolism |
|  |  | alpha-Linolenic acid metabolism |
|  |  | Biosynthesis of unsaturated fatty acids |
|  | 1.4 Nucleotide metabolism | Purine metabolism |
|  |  | Pyrimidine metabolism |
|  | 1.5 Amino acid metabolism | Alanine, aspartate and glutamate metabolism |
|  |  | Glycine, serine and threonine metabolism |
|  |  | Cysteine and methionine metabolism |
|  |  | Valine, leucine and isoleucine degradation |
|  |  | Valine, leucine and isoleucine biosynthesis |
|  |  | Lysine biosynthesis |
|  |  | Lysine degradation |
|  |  | Arginine and proline metabolism |
|  |  | Histidine metabolism |
|  |  | Tyrosine metabolism |
|  |  | Phenylalanine metabolism |
|  |  | Tryptophan metabolism |
|  |  | Phenylalanine, tyrosine and tryptophan biosynthesis |
|  | 1.6 Metabolism of other amino acids | beta-Alanine metabolism |
|  |  | Taurine and hypotaurine metabolism |
|  |  | Selenocompound metabolism |
|  |  | Cyanoamino acid metabolism |
|  |  | D-Glutamine and D-glutamate metabolism |
|  |  | D-Arginine and D-ornithine metabolism |
|  |  | Glutathione metabolism |
|  | 1.7 Glycan biosynthesis and metabolism | N-Glycan biosynthesis |
|  |  | Mucin type O-Glycan biosynthesis |
|  |  | Other types of O-glycan biosynthesis |
|  |  | Glycosaminoglycan biosynthesis - CS/DS |
|  |  | Glycosaminoglycan biosynthesis - HS/Hep |
|  |  | Glycosaminoglycan biosynthesis - KS |
|  |  | Glycosaminoglycan degradation |
|  |  | Glycosylphosphatidylinositol(GPI)-anchor biosynthesis |
|  |  | Glycosphingolipid biosynthesis - lacto and neolacto series |
|  |  | Glycosphingolipid biosynthesis - globo series |
|  |  | Glycosphingolipid biosynthesis - ganglio series |
|  |  | Other glycan degradation |
|  | 1.8 Metabolism of cofactors and vitamins | Thiamine metabolism |
|  |  | Riboflavin metabolism |
|  |  | Vitamin B6 metabolism |
|  |  | Nicotinate and nicotinamide metabolism |
|  |  | Pantothenate and CoA biosynthesis |
|  |  | Biotin metabolism |
|  |  | Lipoic acid metabolism |
|  |  | Folate biosynthesis |
|  |  | One carbon pool by folate |
|  |  | Retinol metabolism |
|  |  | Porphyrin and chlorophyll metabolism |
|  |  | Ubiquinone and other terpenoid-quinone biosynthesis |
|  | 1.9 Metabolism of terpenoids and polyketides | Terpenoid backbone biosynthesis |
|  | 1.10 Biosynthesis of other secondary metabolites | Caffeine metabolism |
|  |  | Butirosin and neomycin biosynthesis |
|  | 1.11 Xenobiotics biodegradation and metabolism | Metabolism of xenobiotics by cytochrome P450 |
|  |  | Drug metabolism - cytochrome P450 |
|  |  | Drug metabolism - other enzymes |
| Group 2 | 3.2 Signal transduction | Ras signaling pathway |
|  |  | Rap1 signaling pathway |
|  |  | MAPK signaling pathway |
|  |  | ErbB signaling pathway |
|  |  | Wnt signaling pathway |
|  |  | Notch signaling pathway |
|  |  | Hedgehog signaling pathway |
|  |  | TGF-beta signaling pathway |
|  |  | Hippo signaling pathway |
|  |  | VEGF signaling pathway |
|  |  | Jak-STAT signaling pathway |
|  |  | NF-kappa B signaling pathway |
|  |  | TNF signaling pathway |
|  |  | HIF-1 signaling pathway |
|  |  | FoxO signaling pathway |
|  |  | Calcium signaling pathway |
|  |  | Phosphatidylinositol signaling system |
|  |  | cAMP signaling pathway |
|  |  | cGMP-PKG signaling pathway |
|  |  | PI3K-Akt signaling pathway |
|  |  | AMPK signaling pathway |
|  |  | mTOR signaling pathway |
|  | 4.3 Cell growth and death | p53 signaling pathway |
|  | 4.4 Cellular community | Signaling pathways regulating pluripotency of stem cells |
|  | 5.1 Immune system | Toll-like receptor signaling pathway |
|  |  | NOD-like receptor signaling pathway |
|  |  | RIG-I-like receptor signaling pathway |
|  |  | Cytosolic DNA-sensing pathway |
|  |  | T cell receptor signaling pathway |
|  |  | B cell receptor signaling pathway |
|  |  | Fc epsilon RI signaling pathway |
|  |  | Chemokine signaling pathway |
|  | 5.2 Endocrine system | Insulin signaling pathway |
|  |  | Adipocytokine signaling pathway |
|  |  | PPAR signaling pathway |
|  |  | GnRH signaling pathway |
|  |  | Estrogen signaling pathway |
|  |  | Prolactin signaling pathway |
|  |  | Oxytocin signaling pathway |
|  |  | Thyroid hormone signaling pathway |
|  | 5.3 Circulatory system | Adrenergic signaling in cardiomyocytes |
|  | 5.6 Nervous system | Retrograde endocannabinoid signaling |
|  |  | Neurotrophin signaling pathway |
| Group 3 | 2.1 Transcription | RNA polymerase |
|  |  | Basal transcription factors |
|  |  | Spliceosome |
|  | 2.2 Translation | Ribosome |
|  |  | Aminoacyl-tRNA biosynthesis |
|  |  | RNA transport |
|  |  | mRNA surveillance pathway |
|  |  | Ribosome biogenesis in eukaryotes |
|  | 2.3 Folding, sorting and degradation | RNA degradation |
|  | 2.4 Replication and repair | DNA replication |
|  |  | Base excision repair |
|  |  | Nucleotide excision repair |
|  |  | Mismatch repair |
|  |  | Homologous recombination |
|  |  | Non-homologous end-joining |
|  |  | Fanconi anemia pathway |
|  | 4.3 Cell growth and death | Cell cycle |
|  |  | Apoptosis |
| Group 4 | 2.3 Folding, sorting and degradation | Protein export |
|  |  | Protein processing in endoplasmic reticulum |
|  |  | SNARE interactions in vesicular transport |
|  |  | Ubiquitin mediated proteolysis |
|  |  | Sulfur relay system |
|  |  | Proteasome |
|  | 3.1 Membrane transport | ABC transporters |
|  | 4.1 Transport and catabolism | Endocytosis |
|  |  | Phagosome |
|  |  | Lysosome |
|  |  | Peroxisome |
|  |  | Regulation of autophagy |
| Group 5 | 3.3 Signaling molecules and interaction | Neuroactive ligand-receptor interaction |
|  |  | Cytokine-cytokine receptor interaction |
|  |  | ECM-receptor interaction |
|  |  | Cell adhesion molecules (CAMs) |
|  | 4.2 Cell motility | Regulation of actin cytoskeleton |
|  | 4.4 Cellular community | Focal adhesion |
|  |  | Adherens junction |
|  |  | Tight junction |
|  |  | Gap junction |
|  | 5.1 Immune system | Hematopoietic cell lineage |
|  |  | Complement and coagulation cascades |
|  |  | Platelet activation |
|  |  | Natural killer cell mediated cytotoxicity |
|  |  | Antigen processing and presentation |
|  |  | Fc gamma R-mediated phagocytosis |
|  |  | Leukocyte transendothelial migration |
|  |  | Intestinal immune network for IgA production |
|  | 5.2 Endocrine system | Insulin secretion |
|  |  | Ovarian steroidogenesis |
|  |  | Progesterone-mediated oocyte maturation |
|  |  | Thyroid hormone synthesis |
|  |  | Melanogenesis |
|  |  | Renin-angiotensin system |
|  | 5.3 Circulatory system | Cardiac muscle contraction |
|  |  | Vascular smooth muscle contraction |
|  | 5.4 Digestive system | Salivary secretion |
|  |  | Gastric acid secretion |
|  |  | Pancreatic secretion |
|  |  | Bile secretion |
|  |  | Carbohydrate digestion and absorption |
|  |  | Protein digestion and absorption |
|  |  | Fat digestion and absorption |
|  |  | Vitamin digestion and absorption |
|  |  | Mineral absorption |
|  | 5.5 Excretory system | Vasopressin-regulated water reabsorption |
|  |  | Aldosterone-regulated sodium reabsorption |
|  |  | Endocrine and other factor-regulated calcium reabsorption |
|  |  | Proximal tubule bicarbonate reclamation |
|  |  | Collecting duct acid secretion |
|  | 5.6 Nervous system | Glutamatergic synapse |
|  |  | GABAergic synapse |
|  |  | Cholinergic synapse |
|  |  | Dopaminergic synapse |
|  |  | Serotonergic synapse |
|  |  | Long-term potentiation |
|  |  | Long-term depression |
|  |  | Synaptic vesicle cycle |
|  | 5.7 Sensory system | Phototransduction |
|  |  | Olfactory transduction |
|  |  | Taste transduction |
|  |  | Inflammatory mediator regulation of TRP channels |
|  | 5.8 Development | Dorso-ventral axis formation |
|  |  | Axon guidance |
|  |  | Osteoclast differentiation |
|  | 5.9 Environmental adaptation | Circadian rhythm |
|  |  | Circadian entrainment |

Supplementary Table S4. **The protein-protein interaction (PPI) databases.**

To construct cellular network models and identify hub-like molecules and nodes with large clustering coefficient (NLCCs), we integrated the six PPI databases as follows. The number of interactions and proteins, and the corresponding references for each database were shown in the table.

|  | **BioGRID** | **CCSB** | **HPRD** | **IntAct** | **MINT** | **DIP** | **Total** |
| --- | --- | --- | --- | --- | --- | --- | --- |
| Number of interactions | 151,432 | 25,769 | 35,377 | 64,018 | 19,259 | 3,866 | 191,822 |
| Number of Proteins | 15,555 | 8,153 | 9,069 | 11,368 | 6,634 | 2,564 | 16,382 |
| Reference | 18 | 19,20,21,22 | 23 | 24 | 25 | 26 | - |

Supplementary Table S5. **The protein-DNA interaction (PDI) databases.**

To identify key TFs targeting DEPs, we integrated the seven PDI databases as follows. The number of interactions and TFs, and the corresponding references for each database were shown in the table

|  | **HTRIdb** | **TRED** | **Amadeus** | **MSigDB** | **EEDB** | **bZIPDB** | **MetaCore™** | **Total** |
| --- | --- | --- | --- | --- | --- | --- | --- | --- |
| Number of interactions | 52,467 | 7,558 | 12,851 | 154,171 | 41,981 | 354 | 62,683 | 304,217 |
| Number of TFs | 284 | 111 | 26 | 286 | 179 | 36 | 918 | 1,019 |
| Reference | 27 | 28 | 29 | 30 | 31 | 32 | GeneGo,  St. Joseph, MI, USA (https://portal.genego.com/) | - |

Supplementary Table S6. **The kinase-substrate interaction databases.**

To identify key kinases targeting DPPs, we integrated the three kinase-substrate interaction databases as follows. The number of interactions and kinases, and the corresponding references for each database were shown in the table.

|  | **PhosphositePlus®** | **Phospho.ELM** | **PhosphoPOINT** | **Total** |
| --- | --- | --- | --- | --- |
| Number of interactions | 4,310 | 3,092 | 2,340 | 5,563 |
| Number of Kinases | 323 | 340 | 264 | 356 |
| Reference | 33 | 34 | 35 | - |

Supplementary Table S9. **Association of signaling pathways in the network model with gastric cancers**

A number of signaling pathways in the network model were previously reported in association with gastric cancers. Experimental systems (cell lines or tissues) in which the associations of the pathways with human gastric cancers were demonstrated, as well as types of complementary coverage related to the pathways (Figure 3), are also shown together with the references.

| **Signaling pathways**  **(Complementary coverage, CC)** | **References** | **Experimental systems** | **Gastric cancer association** |
| --- | --- | --- | --- |
| PPAR signaling pathway (CC1) | Takahashi *et al*., 1999[^1^](#_ENREF_1) | Gastric cancer cell line MKN45 | Activation of PPARг inhibited cell growth and induced apoptosis in gastric cancer cells. |
|  | Hashimoto *et al*., 2004[^2^](#_ENREF_2) | Gastric cancer tissues from patients | A subset of human gastric carcinoma expresses H-FABP, and its expression is associated with disease progression, tumor aggressiveness, and poor patient survival. |
| ErbB signaling pathway (CC1) | Wu *et al*., 2010[^3^](#_ENREF_3) | Gastric cancer patients | EGFR and HER2 can act as molecular targets for the development of pathway-directed cancer therapy. Trastuzumab and Combination of cetuximab and FOLFIRI targeting these targets were reported to show efficacy in gastric cancer patients. |
|  | Zhang *et al*., 2009[^4^](#_ENREF_4) | Gastric cancer tissues from patients | The intestinal type of gastric cancer exhibited a higher rate of HER2 overexpression than the diffuse type, whereas the diffuse type of gastric cancer exhibited the overexpression of HER3. |
|  | TCGA, 2014[^5^](#_ENREF_5) | Primary gastric cancer tissues | Gastric adenocarcinoma was characterized by ErbB signaling-related molecules such as ErbB2 and 3. |
|  | Lang *et al*., 2007[^6^](#_ENREF_6) | Gastric cancer cell lines TMK-1 and KKLS | The strong expression of phospho-mTOR was observed in diffuse-type gastric adenocarcinomas. Also, rapamycin treatment led to significant inhibition of tumor growth, reducing HIF-1α activity. |
|  | Hashimoto *et al*., 2008[^7^](#_ENREF_7) | Gastric cancer cell line NUGC4 | Blocking the CXCR4/mTOR signaling pathway by using rapamycin induced autophagic cell death in gastric cancer. |
| PI3K-Akt signaling pathway (CC2, 3)  and  Phosphatidylinositol signaling system (CC3) | Oki *et al*., 2005[^8^](#_ENREF_8) | Primary gastric carcinoma tissues | AKT phosphorylation led to chemoresistance for gastric cancer. |
|  | Osaki *et al*., 2004[^9^](#_ENREF_9) | Gastric cancer cell line MKN45 | The inhibition of the PI3K-Akt signaling pathway enhanced the sensitivity of Fas-mediated apoptosis. |
|  | Fei *et al*., 2002[^10^](#_ENREF_10) | Gastric cancer tissues from patients | PTEN expression was reduced or absent in large proportion of gastric cancer samples compared with the matched non-malignant gastric biopsy. |
|  | Nakamura *et al*., 2005[^11^](#_ENREF_11) | Gastric cancer tissues from patients | EPHA2 was up-regulated in tumor tissues than adjacent normal tissues. |
| Lysosome (CC1, 3) | Pavelic *et al*., 2003[^12^](#_ENREF_12) | Primary gastric cancer tissues | Insulin-like growth factor 2 receptor, which is involved in lysosomal pathway, was associated with suppression of cancer cell growth. |
|  | Ebert *et al*., 2005[^13^](#_ENREF_13) | Gastric cancer tissues from patients | Active form of cathepsin B was over-expressed in gastric cancer tissues. |
|  | Hippo et al., 2002[^14^](#_ENREF_14) | Gastric cancer tissues from patients | GALC and CTSK genes were up-regulated in gastric cancer tissues. |
| Calcium signaling pathway (CC1, 3) | Sakakura *et al*., 2003[^15^](#_ENREF_15) | Gastric cancer cell lines from malignant ascites | The antagonist of IP3R, 2APB, inhibited cell proliferation and induced apoptosis in gastric cancer cells. |
|  | Zhu et al., 1999[^16^](#_ENREF_16) | Gastric cancer cell line AGS | Non-steroidal anti-inflammatory drug-induced apoptosis in gastric cancer cells was blocked by protein kinase C activation through inhibition of c-myc |
|  | Suzuki et al., 2010[^17^](#_ENREF_17) | Gastric cancer tissues from patients | PDGFR-β was more activated in gastric tumor than normal tissue, and the activation was correlated with both angiogenesis and cancer invasion. |
|  | Piontek et al., 1993[^18^](#_ENREF_18) | Gastric cancer cell line AGS | Prolonged activation of protein kinase C by TPA and activation of adenylate cyclase by forskolin resulted in a dose-dependent growth inhibition of AGS gastric tumor cells |
|  | Ribiczey et al., 2007[^19^](#_ENREF_19) | Gastric cancer cell line KATO-III | PMCA4b(ATP2B4) expression was enhanced during trichostatin A-induced differentiation of gastric cancer cell lines |

# SUPPLEMENTARY METHODS

**Chemicals.** Tris (hydroxymethyl) aminomethane, urea, ammonium bicarbonate, triethylammonium bicarbonate, iodoacetamide and formic acid were purchased from Sigma-Aldrich (St. Luis, MO). Sodium dodecyl sulfate (SDS) and dithiothreitol (DTT) were obtained from USB corporation (Cleverand, OH) and GE Healthcare Life Sciences (Uppsala, Sweden), respectively. Bicinchoninic acid (BCA) protein assay kit was purchased from Pierce (Thermo Scientific, Rockford, IL). Sequence-grade modified trypsin was obtained from Promega (Madison, WI). iTRAQ reagents were purchased from AB Sciex (Foster City, CA).

**Protein extraction and digestion.** After tissues were carefully washed in PBS buffer [137 mM NaCl, 2.7mM KCl, 10mM Na_2_HPO_4_ and 1.8 mM KH_2_PO_4_, pH 7.4 (adjusted with HCl)] on ice to remove blood, the pieces of tumor and adjacent normal tissues were individually cryopulverized using a Cryoprep device (CP02, Covaris). Each tissue piece (66-85 mg in total wet tissue weight) was placed in a cryovial (Covaris, 430487) on dry ice, subsequently transferred to a Covaris tissue bag (TT1, Covaris), and pulverized at an impact level 3 after placing in the tissue bag into liquid nitrogen for 30 sec. The tissue powder from each different tissue was then placed in a sonication tube (Covaris, 002109) and mixed with lysis buffer [4% SDS, 0.1 M Tris-HCl pH 7.6 and one tablet phosphatase inhibitor (PhosSTOP, Roche) in 10 mL]. Different volume of lysis buffer was used depending on the total tissue weight (ca. 1 mL for 20 mg). Tissue lysis was performed by sonication using a focused-ultrasonicator (Covaris, S220) at a setting of 2W (intensity 5) for 5 s followed by 36 W (intensity 10) for 20 s and 0 W (intensity 0) for 10 s. The sonication cycle was repeated for 20 times at 16 °C. The homogenate was centrifuged at 16,000 g and 20°C for 10 min (5810 R, Eppendorf) and the supernatant was transferred to a new tube. The debris was further lysed using a probe sonicator (Q55 Sonicator, Qsonica) and centrifuged at 16,000 g and 20°C for 10 min and the supernatant was combined with the previous one. Protein concentration was then measured using the BCA protein assay (BCA Protein Assay Kit, Pierce).

Ca. 3.5 mg proteins of each tissue type (i.e. tumor or normal) was divided into seven 500 μg portions and each portion was digested using a Filter Aided Sample Preparation (FASP) method separately in seven filter units[^20^](#_ENREF_20). 500 μg proteins was reduced in SDT buffer (4% SDS in 0.1M Tris-HCl, pH 7.6 and 0.1 M DTT) at 37°C for 45 min with shaking at 300 rpm and boiled for 10 min at 95 °C on a thermomixer (Comfort, Eppendorf). The protein sample was then sonicated for 10 min in a bath sonicator (5800, Branson) followed by centrifugation at 16,000 g and 20 °C for 5 min. The supernatant was transferred to a membrane filter (YM-30, Millipore Corporation), in which the protein sample was mixed with 200 μL of 8 M urea (in 0.1 M Tris-HCl, pH 8.5). The protein sample on the membrane filter was centrifuged at 14,000 g and 20 °C for 60 min to remove SDS. This step of SDS removal was repeated three times. Subsequently, 100 μL of 0.05 M iodoacetamide in 8 M urea was added to the filter for alkylation for 25 min at room temperature in the dark, followed by centrifugation at 14,000 g and 20 °C for 30 min. The protein samples on the membrane filters were diluted with 200 μL of 8 M urea and concentrated again. This step was repeated four times. Finally, 100 μL of 50 mM ammonium bicarbonate, pH 8.0, was added to the filter, followed by centrifugation at 14,000 g and 20 °C for 30 min. This step of buffer exchange was repeated twice. Trypsin (Promega; Madison; WI) was added to the filter at an enzyme to protein ratio of 1: 50 (w/w) and the proteins were digested at 37 °C for overnight. After the first digestion, the second digestion was carried out with trypsin (1:100 ratio) at 37 °C for 6 h. The resulting peptides were then eluted by centrifugation at 14,000 g and at 20 °C for 30 min. The filter was rinsed with 60 μL of 50 mM ammonium bicarbonate and centrifuged at 14,000 g and at 20 °C for 20 min. The eluents were combined and dried. The peptide samples were kept in -80 °C until the subsequent iTRAQ labeling.

**iTRAQ labeling.** Peptides were labeled with 4-plex iTRAQ^TM^ reagent (AB Sciex, Foster City, CA). The seven peptide samples from FASP digestion of a normal adjacent tissue were merged into two independent biological replicates (800 μg/replicate) that were labeled with eight units of 114 and 116 iTRAQ reagents, respectively. The seven peptide samples from FASP digestion of a tumor tissue mass were merged into two independent biological replicates (800 μg/replicate) that were labeled with eight units of 115 and 117 iTRAQ reagent, respectively. A total of 32 iTRAQ reactions (4 iTRAQ × 8 peptide units) were proceeded in parallel. Briefly, each 100 μg peptides was dissolved in 30 μL of dissolution buffer (500 mM TEAB, pH 8.5) and a unit of an iTRAQ reagent was dissolved in 70 μL of ethanol. Subsequently, the prepared iTRAQ reagent was transferred to the peptide sample and the mixture was vortexed briefly and incubated for 1 h at room temperature in a thermomixer. At the end of the labeling reaction, the unreacted reagents were hydrolyzed by adding 300 μl of 0.05% TFA and incubating for 30 min at room temperature. Finally, all of the iTRAQ labeled peptides from a patient were pooled and concentrated by vacuum centrifugation. The labeled sample was concentrated to 200 μL and subjected to mid-pH reverse-phase liquid chromatography (mRP-LC) fractionation.

**Mid-pH RP fractionation*.*** The iTRAQ labeled peptides were fractionated and concatenated into 24 fractions as described previously[^21^](#_ENREF_21). Peptides sample were loaded on a guard column (Xbridge, C18 5µm, 4.6 mm
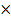
 20 mm) and an analytical column (Xbridge, C18 5µm, 4.6 mm
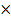
 250 mm) for separation. A gradient was generated using an Agilent 1260 Infinity HPLC system (Agilent, Palo Alto, CA) operated with solvent A (10mM TEAB in water, pH 7.5) and solvent B (10mM TEAB in 90 % ACN, pH 7.5). ca. 3.2 mg iTRAQ-labeled peptides in 200 μL iTRAQ solution were diluted to 900 μL using solvent A and the entire peptide solution was injected. A 115 min gradient with flow rate of 500 μl/min was then applied as follows: holding at 100 % solvent A for 10 min, from 0% to 5% solvent B in 10 min, from 5% to 35 % over 60 min, from 35% to 70 % in 15 min, 70 % in 10 min, and 70% to 0% in 10 min. Every 1 min of the eluent between 15 min and 110 min was sequentially collected into each well of 96-well plate using a fraction collector (G1364C, Agilent) at 4 °C. The 96 well fractions were divided into four different sections according to their elution time: early section (from #1 to #24 well), early mid-section (from #25 to #48 well), late mid-section (from #49 to #72 well) and late section (from #73 to #96 well). The 96 fractions were concatenated into 24 fractions by combining four fractions from each section (i.e., #1-#25-#49-#73, #2-#26-#49-#74, ... #24-#48-#72-#96). The concatenated 24 fractions were vacuum dried and stored at -80 °C until LC-MS/MS experiments.

**Phosphopeptide enrichment.** IMAC method was used to enrich phosphopeptides from 12 fractions obtained by combining adjacent fractions of the 24 fractions (e.g. #1&#2 , #3&#4, and so on.). IMAC bead was prepared from Ni-NTA magnetic bead (36113, Qiagen GmbH). 1.5 mL of Ni-NTA bead slurry was washed three times with 1.2 mL deionized water (DIW) and was then reacted with 1.2 mL of 100 mM EDTA (pH 8.0) by gently mixing for 30 min on an end-over-end rotator (SB3, Stuart) to remove Ni^2+^ ions. After the reaction, EDTA solution was removed, and beads were washed three times with 1.2 mL of DIW. The beads were then reacted with 1.2 mL of freshly prepared 10 mM aqueous FeCl_3_ solution for 30 min with end-over-end rotation. The Fe^3+^-NTA beads were washed three times with 1.2 mL deionized water and resuspended in 1.2 mL of 1:1:1 ACN/MeOH/0.01% acetic acid for aliquoting into 12 microcentrifuge tubes, each containing 100 μL bead solution. Phosphopeptides from each fraction were enriched in batch mode, in which 12 IMAC experiments were performed simultaneously using a 12-tube magnet (36912, Qiagen GmbH). Fe^3+^-NTA beads of each tube was washed with 400 μL binding buffer (80% ACN/0.1% TFA). Each of the iTRAQ labeled peptide sample (ca. 250 μg each) of the 12 fractions was resuspended in 500 μL of binding buffer and was transferred to a tube of the aliquoted beads. The binding reactions were proceeded for 30 min with end-over-end rotation. After the binding reaction, the reacted beads were washed with 500 μL binding buffer for four times. In this step, the flow-throughs were collected and dried for the down-stream N-glycopeptide enrichment. Finally, the bound phosphopeptides were eluted from the beads by incubating in 125 μL of 1:1 ACN/2.5% ammonia in 2 mM phosphate buffer (pH 10) for 1.5 min. The eluted phosphopeptides were acidified immediately with 10% TFA to pH 3.5-4.0 before vacuum dry.

**N-glycopeptide enrichment.** A modified filter aided capture and elution (FACE) protocol was used to enrich N-glycopeptides. The flow-through from the IMAC experiment on each peptide fraction was resuspended in 45 μL of CW lectin mixture (200 μg ConA, and 200 μg WGA in 45 μL of 2× binding buffer (2 mM MnCl_2_, 2 mM CaCl_2_, 1M NaCl and 40 mM Tris-HCl, pH 7.6)). The each sample of 12 fractions was transferred to YM-30 membrane filter and incubated for 1 h at room temperature for lectin binding reaction. After incubation, unbound peptides were removed by centrifugating the filter at 14,000 g and 20 °C for 10 min. The lectin captured peptides were washed with 500 μL binding buffer (1 mM MnCl_2_, 1 mM CaCl_2_, 0.5 M NaCl and 20 mM Tris-HCl pH 7.3) for four times. Subsequently, 125 μL of 40 mM NH_4_HCO_3_ were added to the filter centrifuged at 14,000 g at 20 °C for 20 min. This step of buffer exchange was repeated twice. The PNGase F solution (5 unit of PNGase F (Roche) in 100 μL of 40 mM NH_4_HCO_3_) was added to the filter and incubated for 3 h at 37℃. Finally, the deglycosylated peptides were eluted by centrifugation at 14,000 g and 20 °C for 20 min and the filter was washed with 125 μL of 40 mM NH_4_HCO_3_ twice. The eluents were combined and dried by vacuum centrifugation.

**LC-MS/MS analysis.** All peptide samples were separated on a dual-online UPLC system that was equipped with two analytical columns (75 μm × 100 cm) and two trap columns (150 μm × 3 cm) manufactured in-house by slurry packing of C18 resin (Jupiter, 3 µm, 300 Å, Phenomenex) [^22^](#_ENREF_22). The operation temperature of the analytical columns was at 60 °C. The solvent A and B were 0.1 % formic acid in water and 0.1 % formic acid in acetonitrile, respectively. A 180 min gradient (from 1% to 40% solvent B over 160 min, from 40 % to 80 % solvent B over 5 min, 80 % solvent B for 10 min and 1% solvent B for 5min) was used for the analysis of profiling of global and N-glycoproteome. For the phosphoproteome analysis 240 min gradient (from 1% to 50% solvent B over 220 min, from 50 % to 80 % solvent B over 5 min, 80 % solvent B for 10 min and 1% solvent B for 5min) was used. The flow rate of all experiments was set to 300 nL/min. For global peptide analysis, 10 μg peptide from each of the 24 fractions were individually analyzed. For phosphopeptide and N-glycopeptide analyses, all of the enriched peptides from each of the 12 fractions were injected.

The eluted peptides from LC was mass-analyzed on Q Exactive Orbitrap mass spectrometer (ThermoScientific, Bremen, Germany) that was equipped with a home-built nano-electrospray source. The electric potential of electrospray ionization was kept at 2.4 kV and the temperature of desolvation capillary was set to 250 °C. Full MS scans were acquired for the mass range of 400 – 2000 Th at the resolution of 70,000. Ten most abundant ions were fragmented by data dependent MS/MS experiments with an isolation window of ±0.8 Th, the exclusion duration of 30 s and at a normalized collision energy (NCE) of 30 for higher energy collisional dissociation (HCD). The charge state of 1 was discarded. The MS/MS scans were acquired at a resolution of 17,500 with a fixed first m/z of 100 Th. Maximum ion injection times were 20 ms and 60 ms for full MS and MS/MS scan, respectively. Automated gain control (AGC) target value was set to 1.0 × 10^6^ for both MS and MS/MS scan.

**Peptide and protein identification.** All tandem spectra were created based on mzXML file using msconvert (ProteoWizard release: 3.0.6909). MS data were first analyzed using PE-MMR to assign accurate precursor mass to tandem MS data[^23^](#_ENREF_23). The MS/MS spectra were searched against a composite database of uniprot-human-reference (May, 2013; 90,191 entries) and 179 common contaminants in the target-decoy setting using MS-GF+ (v9387) search engine under the following condition: Semi tryptic, precursor mass tolerance of 10 ppm, carbamidomethylation of cysteine and iTRAQ labeling of lysine and peptide n-termini as static modification and oxidation of methionine as variable modification. For the analysis of phosphopeptides, the variable modifications of phosphorylation to serine/threonine/tyrosine were additionally used. For N-glycopeptide analysis, a variable modification of deamidation to asparagines was additionally used. For global peptide data, the search results of 24 LC-MS/MS data were all combined and the target-decoy analysis was performed on the combined dataset to obtain peptides at the false discovery rate (FDR) 0.01. For phosphopeptide and N-glycopeptide data, the search results of each data set of 12 LC-MS/MS phosphopeptide and N-glycopeptide data were combined for filtering at PSM FDR 0.01 by target-decoy analyses, respectively. After obtaining PSMs at FDR 0.01 for phosphopeptide data, the unique mass class (UMC) filter was used to remove the ambiguity associated with phosphorylation site[^24^](#_ENREF_25). For N-glycopeptide data, the N-glycopeptides having N-X-S/T motif (X: any amino acid except proline) were only used for further analyses.

The peptides with false discovery rates (FDRs) ≤ 0.01 were selected and then clustered into protein groups according to procedure as previously described. For each protein group, constituent proteins were ranked in descending order of their sequence coverages, and the protein with the maximum protein sequence coverage was selected as a representative protein of the protein group, which was mapped to the corresponding gene based on the gene-protein table in Uniport DB. Protein groups of more than two non-redundant peptides were reported and used for the subsequent analyses. The protein FDR is estimated to be 1.4%, on average for the three patients. For the case of phosphopeptide and N-glycopeptide analysis, all protein groups identified by the PTM peptides were used. Representative proteins from protein groups were then mapped to Ensembl 71 mapping table to obtain non-redundant Gene IDs. Some proteins which were not available in Ensembl 71 mapping table were searched for match again against neXtProt (http://nextprot.org, release June 2013).

**References**

1. Takahashi, N. et al. Activation of PPARgamma inhibits cell growth and induces apoptosis in human gastric cancer cells. *FEBS Lett* **455**, 135-139 (1999).

2. Hashimoto, T. et al. Expression of heart-type fatty acid-binding protein in human gastric carcinoma and its association with tumor aggressiveness, metastasis and poor prognosis. *Pathobiology.* **71**, 267-273 (2004).

3. Wu, W.K. et al. Dysregulation of cellular signaling in gastric cancer. *Cancer Lett* **295**, 144-153 (2010).

4. Zhang, X.L. et al. Comparative study on overexpression of HER2/neu and HER3 in gastric cancer. *World J. Surg.* **33**, 2112-2118 (2009).

5. Cancer Genome Atlas Research, N. Comprehensive molecular characterization of gastric adenocarcinoma. *Nature* **513**, 202-209 (2014).

6. Lang, S.A. et al. Mammalian target of rapamycin is activated in human gastric cancer and serves as a target for therapy in an experimental model. *Int. J. Cancer* **120**, 1803-1810 (2007).

7. Hashimoto, I. et al. Blocking on the CXCR4/mTOR signalling pathway induces the anti-metastatic properties and autophagic cell death in peritoneal disseminated gastric cancer cells. *Eur. J. Cancer* **44**, 1022-1029 (2008).

8. Oki, E. et al. Akt phosphorylation associates with LOH of PTEN and leads to chemoresistance for gastric cancer. *Int. J. Cancer* **117**, 376-380 (2005).

9. Osaki, M. et al. Inhibition of the PI3K-Akt signaling pathway enhances the sensitivity of Fas-mediated apoptosis in human gastric carcinoma cell line, MKN-45. *J. Cancer Res. Clin. Oncol.* **130**, 8-14 (2004).

10. Fei, G. et al. Reduced PTEN expression in gastric cancer and in the gastric mucosa of gastric cancer relatives. *Eur. J. Gastroenterol. Hepatol.* **14**, 297-303 (2002).

11. Nakamura, R. et al. EPHA2/EFNA1 expression in human gastric cancer. *Cancer Sci.* **96**, 42-47 (2005).

12. Pavelic, K. et al. Gastric cancer: the role of insulin-like growth factor 2 (IGF 2) and its receptors (IGF 1R and M6-P/IGF 2R). *J. Pathol.* **201**, 430-438 (2003).

13. Ebert, M.P. et al. Overexpression of cathepsin B in gastric cancer identified by proteome analysis. *Proteomics* **5**, 1693-1704 (2005).

14. Hippo, Y. et al. Global gene expression analysis of gastric cancer by oligonucleotide microarrays. *Cancer Res.* **62**, 233-240 (2002).

15. Sakakura, C. et al. Possible involvement of inositol 1,4,5-trisphosphate receptor type 3 (IP3R3) in the peritoneal dissemination of gastric cancers. *Anticancer Res.* **23**, 3691-3697 (2003).

16. Zhu, G.H. et al. Non-steroidal anti-inflammatory drug-induced apoptosis in gastric cancer cells is blocked by protein kinase C activation through inhibition of c-myc. *Br. J. Cancer* **79**, 393-400 (1999).

17. Suzuki, S. et al. Clinicopathological significance of platelet-derived growth factor (PDGF)-B and vascular endothelial growth factor-A expression, PDGF receptor-beta phosphorylation, and microvessel density in gastric cancer. *BMC Cancer* **10**, 659 (2010).

18. Piontek, M., Hengels, K.J., Porschen, R. & Strohmeyer, G. Protein kinase C and adenylate cyclase as targets for growth inhibition of human gastric cancer cells. *J Cancer Res. Clin. Oncol.* **119**, 697-699 (1993).

19. Ribiczey, P. et al. Isoform-specific up-regulation of plasma membrane Ca2+ATPase expression during colon and gastric cancer cell differentiation. *Cell Calcium* **42**, 590-605 (2007).

20. Wisniewski, J.R., Zougman, A., Nagaraj, N. & Mann, M. Universal sample preparation method for proteome analysis. *Nat. Methods.* **6**, 359-362 (2009).

21. Wang, Y. et al. Reversed-phase chromatography with multiple fraction concatenation strategy for proteome profiling of human MCF10A cells. *Proteomics* **11**, 2019-2026 (2011).

22. Lee, H. et al. A fully automated dual-online multifunctional ultrahigh pressure liquid chromatography system for high-throughput proteomics analysis. *J. Chromatogr. A* **1329**, 83-89 (2014).

23. Shin, B. et al. Postexperiment monoisotopic mass filtering and refinement (PE-MMR) of tandem mass spectrometric data increases accuracy of peptide identification in LC/MS/MS. *Mol. Cell. Proteomics* **7**, 1124-1134 (2008).

24. Madar, I.H. et al. Reduction of Ambiguity in Phosphorylation-site Localization in Large-scale Phosphopeptide Profiling by Data Filter using Unique Mass Class Information. *B. Korean Chem. Soc.* **35**, 845-850 (2014).
